# Influence of short-term chronic oral cannabidiol application on muscle recovery and performance after an intensive training protocol - a randomized double-blind crossover study

**DOI:** 10.1080/15502783.2024.2337252

**Published:** 2024-04-04

**Authors:** Eduard Isenmann, Sebastian Veit, Ulrich Flenker, Alessio Lesch, Dirk W. Lachenmeier, Patrick Diel

**Affiliations:** a German Sport University Cologne, Department of Molecular and Cellular Sports Medicine, Institute for Cardiovascular Research and Sports Medicine, Cologne, Germany; b IST Hochschule of Applied Sciences, Department of Fitness and Health, Dusseldorf, Germany; c Chemical and Veterinary Investigation Agency (CVUA), Karlsruhe, Germany

**Keywords:** Cannabidiol, CBD, muscle damage, recovery, regeneration, performance

## Abstract

**Background:**

Rapid regeneration after intense exercise is essential for competitive athletes. Based on this assumption, supplementation strategies, focusing on food supplements, are increasing to improve the recovery processes. One such supplement is cannabidiol (CBD) which is gaining more attention in competitive sports. However, the evidence is still lacking and there are no data available about the effect of a short-term chronic application.

**Methods:**

A three-arm double-blind cross-over study was conducted to determine the effects of two different CBD products on performance, muscle damage and inflammatory processes in well-trained athletes. In total 17 subjects took successfully part in this study. Each subject underwent the six-day, high-intensity training protocol three times. After each training session, each subject took either a placebo or a CBD product (60 mg of oil or solubilisate). Between the intervention phases, at least four weeks of washout period was conducted. Before and after the training protocols the performance capacity in countermovement jump (CMJ), back squat (BS), bench press (BP) and 1-mile run were measured and biomarkers for muscle damage (creatine kinase, myoglobin), inflammatory processes (interleukin 6 and 10) and immune cell activity (ratios of neutrophil granulocytes, lymphocytes and, platelets) were analyzed. For statistical analyses, the current version of R and a linear mixed model was used.

**Results:**

It could identify different effects of the training protocol depending on performance level (advanced or highly advanced athletes) (*p* < .05). Regardless of the performance level, muscle damage and a reduction in performance could be induced by the training protocol. Only CBD oil was associated with a reduction in myoglobin concentration (*p* < .05) in advanced athletes. Concerning immune activity, a significant decrease in platelets lymphocyte ratios was observed in advanced athletes after placebo treatment (*p* < .05). CBD oil application showed a slight inhibitory effect (*p* < .10). Moreover, the reduction in performance differs between the performance levels. A significant decrease in CMJ was observed in advanced athletes and a decreasing trend in BS was observed in highly advanced athletes after placebo treatment (*p* < 0.10). Both CBD products do not affect performance parameters. For inflammatory parameters, no effects were observed.

**Conclusion:**

It was found that the performance level of the subjects was a decisive factor and that they responded differently to the training protocol and the CBD application. However, no clear effects of either CBD product were found and further research is needed to identify the long-term effects of CBD application.

## 1. Introduction

Systematic training stimuli as well as intensive competition loads disrupt physiological homeostasis, which can lead to muscle damage and subsequent inflammatory reactions and results in reduced performance [1]. Rapid regeneration after intense exercise is therefore essential to maximize the next training stimulus as quickly as possible with maximum performance. Nutritional supplements are often used by athletes to promote regeneration after intensive exertion [2]. It seems that there are even differences between sports and that strength-oriented athletes tend to use nutritional supplements more than other athletes [3]. As a result, interest in cannabidiol (CBD) products has been increasing, not only in recreational but also in competitive sports [4–7]. CBD is one of over 100 phytocannabinoids [8,9] found in *Cannabis sativa*, a plant of the *Cannabaceae* family [10]. CBD, unlike ∆^9^-tetrahydrocannabinol (THC), acts as an inverse agonist and negative allosteric modulator at both cannabinoid receptors (CB1, CB2) [11–13]. Therefore, psychotropic effects are absent even at very high doses [11,14]. Since 2018, the World Anti-Doping Agency (WADA) has taken CBD off the banned list [15], making CBD the only cannabinoid that may be used in competitive sports [16]. This is mainly because CBD has no psychotropic effect. This has been shown in a few human studies, even if using very high doses of up to 6,000 mg [17].

Due to the varied binding affinities of CBD, several studies have been conducted on the effects of CBD in a medical context. Especially based on potential anti-inflammatory effects, positive effects have been demonstrated in diseases such as rheumatoid arthritis [18], colitis [19], diabetes [20] and psoriasis [21]. While the importance of sleep for regeneration is undisputed [22], the effects of CBD on sleep are partly controversial. While an improvement in sleep has been shown in animal models [23,24], these effects could mostly not be replicated in humans [25,26]. However, results from animal models or clinical studies are only suitable to a limited extent for concluding the effectiveness of competitive sports.

Currently, there are only a small number of studies on the influence of CBD on performance in humans.

In addition, studies have primarily only focused on the acute effects of CBD application on performance and regeneration capacity. The effects of short-term or chronic application, in contrast, are very limited.

One study was conducted in the context of endurance performance [27]. Sahinovic et al. [27] compared in a placebo-controlled double-blind study in a crossover design the influence of 300 mg CBD (application: 90 min before the test) on physiological and psychological factors after 60 min of running at 70% of VO_2_ max. Although significant effects of CBD on oxygen uptake and subjective well-being could be demonstrated, the authors put the result into perspective based on the effect size and the sample size. In addition, the authors were unable to determine any influence of either the exercise protocol or CBD application on the pro-inflammatory biomarkers interleukin-6 (IL-6) and tumor necrosis factor α (TNF-α).

In contrast, more studies were conducted concerning CBD and strength training. Cochrane-Snyman et al. [28] investigated the daily intake of CBD (150 mg/day for 3 days, divided into two doses of 75 mg each) during the recovery process after eccentric training. In a double-blinded crossover study, 13 untrained men completed 6 sets of 10 isokinetic-eccentric biceps curls. In addition, the maximum torque during an isometric biceps curl with a joint angle of 115°, the subjective feeling of fatigue, the elbow angle of the relaxed, hanging arm and the arm circumference were measured before and 24, 48 and 72 h after the exercise. Significant changes due to the training were only found in subjective fatigue perception and joint angle, but with no differences between the groups. Crossland and colleagues also investigated the influence of CBD on strength capacity as well as on muscle damage [29]. In a randomized controlled double-blind crossover study, 24 NCAA Division 1 and 2 female athletes performed 10 sets of 10 repetitions of isokinetic eccentric leg extensions (30°/s) twice 30 days apart, with 1 min rest between sets. At baseline and 4 h, 24 h and 48 h after exercise, vertical jump, isometric and dynamic maximal leg extension strength was measured. In addition, myoglobin (MYO), IL-6, interleukin-1β (IL-1ß) and interleukin-10 (IL-10) concentrations were determined at the same measurement times. Two hours before, immediately after and 10 h after the fatigue protocol, subjects consumed either 5 mg/kg BW CBD (224–408 mg per application) or a PLA preparation. At no time was there a significant increase in inflammatory markers or a difference between PLA and CBD. MYO was significantly increased in both groups after 4 h, but not after 24 h and 48 h. A group difference could not be found. Isometric and dynamic maximum strength (60°/s) were significantly reduced after 4 h and 24 h without any difference between the groups. In contrast, significant effects of 60 mg CBD on muscle damage were observed in two independent studies by our research group [31, 32]. In a randomized controlled pilot project in a crossover design with very well-trained athletes (squat performance > 150% of BW [30]), significantly lower creatine kinase (CK) concentrations were found 24 h after exercise following intensive strength training. The subjects performed 3 sets of 12 repetitions of the squat at an intensity of 70% of 1-RM followed by 3 sets of 15 low jumps [31]. However, strength capacity was lower in the CBD group than in the placebo group. In contrast, in well-trained athletes (squat performance: 120–150% of BW [30], significant differences were found only 72 h after exercise with the same training protocol. In a six-arm randomized controlled crossover study, the influence of a single 60 mg CBD application on muscle damage and strength capacity 24 h, 48 h and 72 h after exercise was examined [32]. Based on the available evidence it is likely that the performance level, as well as the training protocol, play a decisive role in increasing muscle damage, inflammatory reactions and the reduction of performance as well as the influence of CBD application.

In contrast to existing studies to the acute effects of CBD on recovery and performance, only one study has yet been conducted on the chronic application of CBD on performance [33]. However, the investigation by Flores et al. only examined the application of 50 mg CBD per day on performance without a training protocol. The authors found no differences between the groups regarding aerobic fitness, muscular strength, physical activity, cognitive health and psychological wellbeing. However, data on chronic application of CBD combined with a training protocol are not available. Consequently, this study aims to examine the influence of short-term chronic CBD application on muscle damage, inflammatory reactions and performance in competitive athletes after an intense training week.

## 2. Material and methods

### 2.1. Subjects

A total of 27 subjects (*m* = 25, w = 2) were recruited for the study. Subjects who were ill, injured or dependent on medication were excluded. The consumption of food supplements or cannabinoids of any kind during and between the interventions was prohibited. All subjects were healthy, between 18 and 40 years old and had an advanced strength level [30]. Men had a relative squat performance of over 120% and a relative bench press performance of over 100% of their body weight (BW). For women, the thresholds were 100% and 60% of their BW. The entire study was conducted during the SARS-CoV-2 pandemic, which is why 8 subjects had to cancel the study early due to illness and subsequent drop in performance or other limitations. 19 subjects completed the study, although two had to be subsequently excluded from the analysis. One subject was unable to replicate the specified minimum performance after the first test, and the other subject had not adhered to the study design and had exercised the day before the entrance tests. Thus, in conclusion, 17 subjects (*m* = 15, f = 2) were included in the analysis.

### 2.2. Study design

A “shock microycle” were carried out for this study with three strength and three endurance sessions. [34,35]. In detail, the aim was to examine the effect of two different CBD preparations (oil and solubilisate (solu)) on the ability to regenerate. For this purpose, a randomized double-blind study was conducted in a triple crossover design. The subjects underwent a six-day exercise protocol three times and consumed either one of the two CBD or a placebo preparation on all days. A four-week washout period was intended to ensure that the CBD accumulated over the intervention would be completely eliminated. The overall study design meets the guidelines for intervention studies [36] and is based on previous investigations [37].

**Figure 1.**
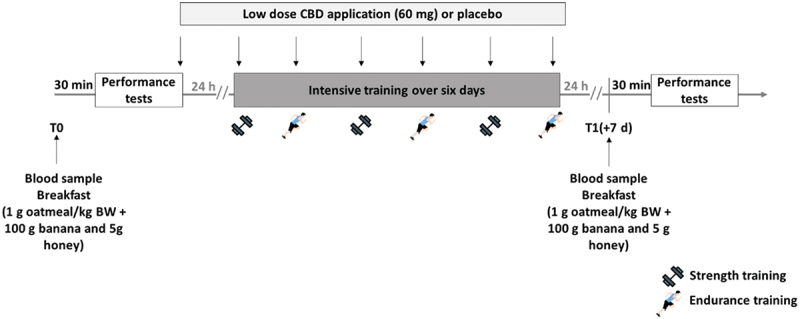
Overall study design.

For the purpose of examining recovery capacity, performance was tested at the beginning of each intervention arm and the day after the last training session, and blood samples were taken. The overall study design can be seen in Figure 1.

### 2.3. Intervention procedure

The subjects arrived fasting at the German Sport University Cologne in the morning (7.00–11.00 am). The entire test procedure took approximately 150 min and had the following schedule:

1. Conducting a COVID-19 rapid test.
2. Blood collection (7.15 – 9.15 am)
3. Determination of the daily body weight
4. Standardized breakfast (1 g/kg BW oatmeal, 100 g banana, 5 g honey, infused with hot water)
5. Digestion phase: 30 min
6. Standardized warm-up (5 min running +5 min dynamic stretching)
7. Jumping Test: (countermovement jump)
8. Maximum strength test (back squat and bench press)
9. Endurance performance test (1600 m run)
10. Taking the first dose of the supplement (only at T0)

**Figure 2.**
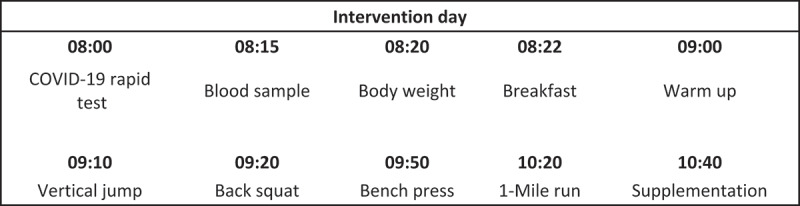
Intervention day.

To exclude adverse effects on the test results, no physical activity was allowed in the 24 h before T0. Only low-intensity physical activity was allowed 48 h before T0. No additional exercise was allowed during the study period (T0 - T7). The time course of the test protocol is shown in Figure 2 with a theoretical start time of 08:00 h. All performance tests are established methods for testing performance according to the NSCA guidelines [38]. Besides all biomarkers are established for identify muscle damage, inflammatory processes and oxidative stress [39–41].

### 2.4. Performance parameters

#### 2.4.1. Counter movement jump

To assess the strength development capacity and approximate the maximum strength of the leg and hip extensor muscles, the maximum jump height during the Counter Movement Jump (CMJ) was measured using an OptoJump Next (Microgate USA, Mahopac (NY), USA) [42,43]. After two warm-up sets, three jumps were measured, the mean value of which was used for the evaluation. The resting time between the jumps was set at 60 s.

One repetition maximum back squat (1-RM BS) and bench press (1-RM BP)

The maximum strength of the lower body was determined with the deep back squat. In the deep squat position, the hip crease must be visually recognizable below the knee crease. A complete movement was declared when the knee and hip angle were fully open at the beginning and the subjects was able to bend into the depth (femur parallel to the floor, 90° knee angle) for a short moment and then fully open the knee and hip angle afterward. The back squat is a valid method for testing the strength performance in lower body [32,37,44,45].

For the upper body, the one repetition maximum in bench press was determined. Lying on a flat bench, the subjects had to lower a loaded barbell onto the chest with the arms flexed and then lift it again until both arms were fully extended. The weight of the barbell was not allowed to be transferred to the chest during chest contact but had to continue to be carried by the upper extremities. The use of the chest as a dynamic element was prohibited. The bench press has also been scientifically proven to be reliable and valid [46] and is therefore used in many studies to determine the maximum strength of the upper extremities [44,47,48].

For both exercises, a standardized warm-up and test protocol were selected, which has already been used in other studies [32,37,44] and are in line with the NSCA guidelines [38]. For each exercise, four warm-up sets were performed with a decreasing number of repetitions and increasing weight, each with a 2 min break in between (10 × 50 %; 8 × 60–70 %; 4–8 × 70–80 %; 2–4 × 80–90 %). After a further 3 min break, the first 1RM test was performed at 90%. After a break of 4 min each, another test was performed with 2.5–10 kg more until concentric muscle failure occurred. The extent of the weight increase depended on the difficulty of the test. The final increase was always by 2.5 kg. The first input test of each subject was based on a 1 RM estimated by the subject and the second and third was each based on the input value of the previous test. The initial test was always identical to the respective initial test to avoid the influence of a different warm-up and weight selection and a different choice of weight for the test trials.

### 2.5. Endurance performance − 1-mile run

Endurance performance was determined by performing a 1,600 m run (1-mile). The running time has a high correlation with the maximum oxygen uptake with an R^2^ value of 0.74 [49] and is therefore used in science to approximate this [50–52]. A standardized warm-up of two rounds of 800 m was performed. Subsequently, the subjects ran four laps ( = 1,600 m) on a standardized 400 m tartan track for time.

### 2.6. Biomarkers

In addition to performance, two 8 ml venous blood samples were taken. Various parameters of muscle damage, inflammation reactions, oxidative stress and immune cell reaction were examined.

#### 2.6.1. Muscle damage: Creatine Kinase (CK) and Myoglobin (MYO)

For muscle damage, the creatine kinase and myoglobin concentrations were analyzed before (T0) and the day after the training week (T1). The CK and MYO serum concentrations were determined with the COBAS h 232 point-of-care system (RocheDiagnostic Systems, Rotkreuz, Switzerland).

#### 2.6.2. Serum Cytokine levels: Interleukin 6 (IL-6) and Interleukin 10 (IL-10)

IL-6 concentrations of serum samples were analyzed using the Human IL-6 ELISA Kit High Sensitivity (Abcam, Cambridge, United Kingdom). IL-10 serum concentrations were analyzed using ELISA Kit High Sensitivity (Abcam, Cambridge, United Kingdom).

#### 2.6.3. Oxidative stress: Oxidative Low-Density Lipoprotein (OxLDL) and Total anti-Oxidative Capacity (TOC)

OxLDL concentrations of serum samples were analyzed using the Human oxLDL/MDA Adduct Elisa. (Immundiagnostik, Bensheim, Germany). For analyzing the TOC concentration of serum concentration, the Human TOC Elisa Kit ImAnOx (TAS/TAC) (anti-oxidative capacity) were used (Immundiagnostik, Bensheim, Germany).

#### 2.6.4. Hematology: immune cell activity

Blood samples were analyzed for routine complete blood count (CBC) including total white blood cells (WBC) count and differential for WBCsub fractions using a BC2300 hematology analyzer (Mindray Medical International Systems, Shenzhen, China).

To identify immune cell activity, the neutrophil granulocyte/lymphocyte (NLR), platelet/lymphocyte and systemic immune-inflammation index (SII) ratios were calculated [53].

### 2.7. Cannabidiol products

Two different CBD products (solubilisate and oil) were used and compared with a placebo preparation (PL). Both CBD products contained 60 mg CBD in the consumption unit and were manufactured for the study (Athenion GmbH, Berlin, Germany). All subjects consumed 60 mg of each of the three preparations directly after their training sessions. In addition, 60 mg was also consumed once after the initial examination. In total, one of the preparations was consumed seven times. Both the subjects and the study coordinators were blinded over the entire period.

#### 2.7.1. CBD solubilisate (Solu)

The CBD-Solu was produced in specific capsules (caps) with a total content of 3 ml (1 ml = 20 mg CBD). The CBD-Solu was dissolved in 200-300 ml of water and drunk as quickly as possible.

#### 2.7.2. CBD-Oil

The CBD oil was filled in eppendorf tubes with a total content of 1 ml. The CBD oil was kept under the tongue for 2 min and then ingested.

#### 2.7.3. Placebo product

The placebo was produced by the same company that manufactured the two CBD products. The placebo was filled into both caps and eppendorf tubes. The placebo had the same color, consistency and taste as the two CBD products, but contained no cannabinoids or other active substances.

To ensure that there was no difference between the three preparations, the subjects were given both caps and eppendorf tubes at each intervention phase, which only differed in their color. Table 1 shows the allocation of the treatments and the ingredients of the storage forms.

**Table 1.** Allocation of CBD products to the treatment groups.

Treatment Group	Caps	Eppendorf Tubes
CBD-Solu	CBD-Solu	Placebo
CBD-Oil	Placebo	CBD-Oil
Placebo	Placebo	Placebo

### 2.8. CBD analyses

To verify the purity of the supplements used, all three products were analyzed by two independent laboratories using mass spectrometry. The eppendorf tubes of all treatments were analyzed by the Institute of Biochemistry of the German Sport University Cologne. The analysis was performed analogously to previous sample analyses [54,55] and the WADA standard [56,57] using gas chromatography/tandem mass spectrometry. In addition, the Chemical and Veterinary Investigation Agency (CVUA) Karlsruhe tested the CBD supplements using validated methodologies (CBD-Solu in the caps and CBD-Oil in the eppendorf tubes). The CBD content was determined by nuclear magnetic resonance spectroscopy [58] and the concentrations of Δ^8^-THC and Δ^9^-THC were determined by high-performance liquid chromatography/tandem mass spectrometry [59].

### 2.9. Training protocol

On the six training days between PRE (T 0) and POST (T 7), a high-intensity training protocol was performed that included whole-body strength training on days 1, 3 and 5, and high-intensity interval training on days 2, 4 and 6 (Table 2). The overall study design followed scientific guidelines and preliminary studies [59–61] to ensure adequate fatigue symptoms. The strength training was deliberately high-frequency [62] and high-volume [63] and performed very close to muscle failure to provoke exercise induced muscle damage (EIMD) as well as strength loss [64–66]. The rest periods between sets were chosen so that conditional and central nervous fatigue was limited so that the subjects could complete the prescribed repetitions in each set. If a subject was unable to perform all the repetitions in one go, the weight was allowed to be taken off for 30 s so that they could then perform the remaining repetitions of the set [67–69]. This meant that the specified number of repetitions could always be performed by all subjects. The total number of 18 work sets per muscle group per week is very high and borders on the range in which some athletes enter overtraining over a longer period [70]. In addition, a high-intensity running protocol [60,61,71,72] was chosen, to produce a high level of fatigue and further muscular damage [71,73,74]. Based on the fact that intensive strength training was combined with high-intensity running, the intervals should be performed as quickly as possible. As a result, no individual heart rate or speed was specified for the participants. Only a minimum distance of 800-1000 m for the men and 600-800 m for the women per interval was specified. This corresponds to a running speed of at least 12 km/h for men and 9 km/h for women per interval. It was pointed out that all intervals must not be completed at the lower limit of the distance. All training sessions were documented by the participants and were evaluated directly after each intervention phase.

**Table 2.** Training protocol.

		Training days 1, 3 and 5		
**Exercise**	Sets	Repetitions	Intensity	Rest
Back Squat	3	10	70 %	150 s
Bench press	3	10	70 %	150 s
Alt. Reverse Lunges	3	12/Leg	35 %	150 s
Dips	3	Max Effort	BW	150 s
Bent over Row	3	12	65 %	150 s
		**Training days 2,4 and 6**		
**Exercise**	Sets	Duration	Intensity	Rest
Running	4	4 min	Max Effort	4 min

alt.: alternating; BW = Body weight

### 2.10. Statistical analyses

All raw data were analyzed using the statistical software R version 4.0.4 (R Core Team, 2021). All performance parameters (CMJ, SQ, BP and mile) were analyzed directly. For the biochemical parameters, a Q-Q plot was used to compare the empirical and theoretical quantiles. In case of linear behavior, the absolute values were used (IL−6, IL−10, PLR, SII). Otherwise, the values were transformed into their natural logarithm (log) (CK, MYO, Ox LDL, InAnOx, NLR). At baseline, ANOVA was used to test the influence of performance level (PL) and a systematic long-term trend over time (ns(Dt, 2)) and its interaction between PL and time (PL : time). As a result, subjects were classified into advanced (Ad) (*n* = 8) and highly advanced (Hi) (*n* = 9) athletes according to Santos [30].

The data were analyzed individually for all test parameters using linear mixed-effects models (LME) [75], with the test parameters always representing the dependent variable. The performance level (PL: Ad or Hi), time (Time: PRE or POST), treatment (Treat: PLA, CBD-Oil, CBD-Solu), as well as the individual effect of time (Dt), represent the independent variables. The individual change over time (Dt) was mapped as a natural 2nd-order spline to correct for long-term adjustment effects and additionally included as a continuous 1st-order autoregressive term as a covariate in the model. Since it had to be assumed that test scores and time are autocorrelated, it was tested whether Dt contributes to general trends and thus represents a significant fixed effect. The model comparisons are based on the likelihood function and changes in the Akaike Information Criterion (AIC). As the potential effects of CBD were to be investigated, the changes of the two groups (Ad & Hi) under PLA conditions from PRE to POST were always analyzed first (PLAd : TimePost & PLHi : TimePost). Subsequently, the influence of CBD products on this change was examined (CBD-Oil: PLAd : TimePost : Treat-Epi & PLHi : TimePost : TreatEpi; CBD-Solu: PLAd : TimePost : TreatCaps & PLHi : TimePost : TreatCaps). If no significant influence of the CBD products on this change could be found, the significance of the temporal change in the PLA condition was assumed analogously for the CBD groups.

The significance level was set at *p* < .05. Effect sizes (ES) were calculated according to Rosenthal and Rosnow [76] as ES = $\frac{2*t}{\surd \mathrm{DF}}$, where t is the t-test statistic and DF is the corresponding degrees of freedom. Effect sizes were classified according to Cohen [77] as trivial (ES < 0.2), small (0.2<ES < 0.5), medium (0.5<ES < 0.8) or large (ES > 0.8) and calculated only for significant group differences. In addition, a Pearson correlation analysis of the pretest performance levels of CMJ, BS, BP and the changes in the blood parameters CK, Myo, GOT, GPT, and GGT was conducted.

## 3. Results

### 3.1. Counter movement jump

**Figure 3.**
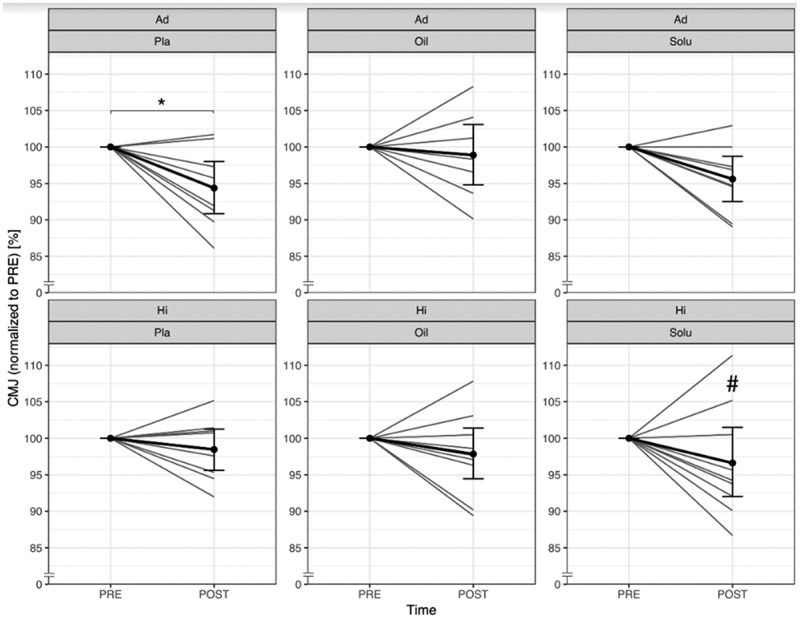
Comparison of normalised counter movement jump values from PRE to POST, divided by group and treatment (grey lines represent the individual courses).

The jump height of the Ad group decreased significantly with all treatments from PRE to POST (*p* = 0.015). Neither CBD-Oil (*p* = 0.221) nor CBD-Solu (*p* = 0.692) had a significant influence on the change. In the Hi group, no significant difference could be detected between PRE and POST (*p* = 0.341). However, a significant group effect could be found with CBD-Solu in comparison to PLA (*p* = 0.016). The normalized values can be found in Figure 3 and differences between the PRE and POST of each subject in supplemental material (Figure 3b).

Ad = Advanced; Hi = Highly-advanced; Pla = Placebo; Oil = CBD-Oil; Solu = CBD-Solu. Ad-Pla: *n* = 8; Ad-Oil: *n* = 7; Ad-Solu: *n* = 8; Hi-Pla: *n* = 8; Hi-Oil: *n* = 9; Hi-Solu: *n* = 9.

### 3.2. 1-RM back squat

**Figure 4.**
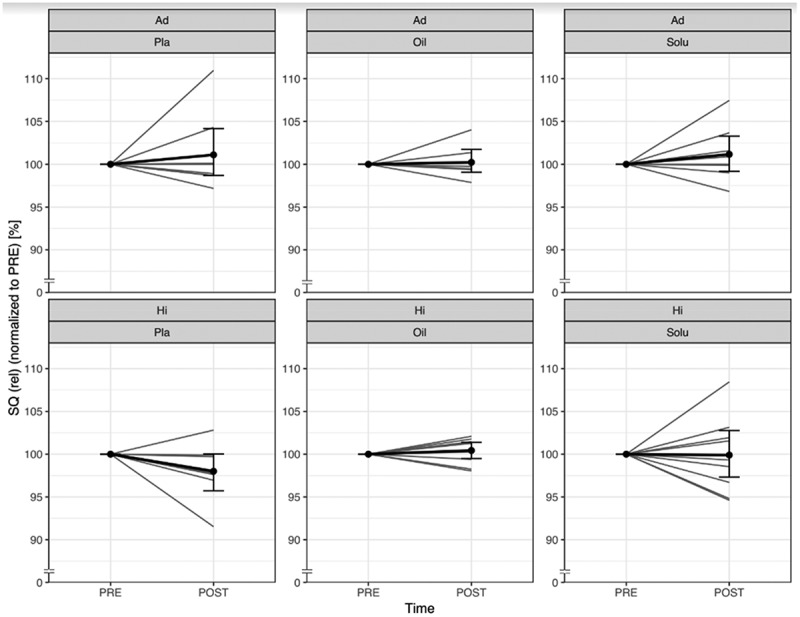
Comparison of normalised back squat values from PRE to POST, divided by group and treatment (grey lines represent the individual courses).

No significant changes from PRE to POST could be found in the absolute and relative performance of the squat (absolute: Ad: *p* = 0.423; Hi: *p* = 0.072; relative: Ad: *p* = 0.465; Hi: *p* = 0.064). Both absolute and relative values show a trend in the Hi group under PLA conditions, with 7 subjects showing a relative loss of performance (Figure 4). Neither the CBD-Oil (Ad: *p* = 0.437; Hi: *p* = 0.261) nor the CBD-Solu (Ad: *p* = 0.457; Hi: *p* = 0.089) influences the change in absolute values. This is confirmed in the relative values (oil = Ad: *p* = 0.643; Hi: *p* = 0.551; Solu = Ad: *p* = 0.591; Hi: *p* = 0.147). The individual differences between pre and post are shown in supplemental material (Figure 4b).

Ad = Advanced; Hi = Highly-advanced; Pla = Placebo; Oil = CBD-Oil; Solu = CBD-Solu. Ad-Pla: *n* = 8; Ad-Oil: *n* = 7; Ad-Solu: *n* = 8; Hi-Pla: *n* = 8; Hi-Oil: *n* = 9; Hi-Solu: *n* = 9.

### 3.3. 1-RM bench press

Absolute bench press performance did not change significantly at any time point (Ad: *p* = 0.900; Hi: *p* = 0.343). Neither CBD-Oil (Ad: *p* = 0.078; Hi: *p* = 0.260) nor CBD-Solu (Ad: *p* = 0.189; Hi: *p* = 0.108) influenced the bench press performance. The relative values also showed no change from PRE to POST (Ad: *p* = 0.956; Hi: *p* = 0.443) and there is also no influence of CBD-Oil (Ad: *p* = 0.449; Hi: *p* = 0.608) or CBD – Solu (Ad: *p* = 0.621; Hi: *p* = 0.260) on relative performance. The normalized changes and the differences between pre and post are shown in supplemental material (Figure 4C,D).

### 3.4. 1600-meter run (1-mile)

In the 1600 m run, neither significant differences over time (Ad: *p* = 0.190; Hi: *p* = 0.750) nor an influence by CBD – oil (Ad: *p* = 0.620; Hi: *p* = 0.580) or CBD-Solu (Ad: *p* = 0.890; Hi: *p* = 0.440) could be detected. The normalized values differences between the PRE and POST of each subject can be found in supplemental material (Figure 4e,f).

All absolute and relative performance values are summarized in Table 3

**Table 3.** Means ± standard deviations separated by performance level, as well as differences (Δ) between PRE and POST, levels of significance (sign.) and effect sizes (ES) of all treatments for bodyweight, countermovement jump, back squat (absolute & relative), bench press (absolute & relative) and 1 mile run.

		Placebo					CBD-Oil					CBD-Solu				
Parameter	Unit	Group	PRE	POST	Δ	Sign.	PRE	POST	Δ	Sign.	ES	PRE	POST	Δ	Sign.	ES
Body Weight	[kg]	Ad	83.7 ± 11.7	83.9±12.0	0.2		84.0 ± 12.2	85.0 ± 13.4	1.0			84.4 ± 12.3	84.5 ± 11.8	0.10		
		Hi	87.5 ± 7.0	87.8 ± 7.0	0.3		86.7 ± 5.6	86.9 ± 5.6	0.1			87.7 ± 6.3	87.0 ± 7.1	−0.7		
CMJ	[cm]	Ad	36.7 ± 5.0	34.5 ± 4.4	−2.2	*	34.9 ± 4.7	34.4 ± 3.8	−0.5			36.7 ± 4.8	35.0 ± 4.4	−1.7		
		Hi	43.5 ± 8.1	42.7 ± 7.1	−0.8		42.4 ± 6.9	41.2 ± 5.2	−1.2			42.4 ± 7.6	40.7 ± 6.0	−1.7	#	−0.81
Back Squat	[kg]	Ad	110.0 ± 12.2	111.2 ± 11.3	1.2		110.7 ± 13.0	112.1 ± 14.0	1.4			111.6 ± 13.1	113.0 ± 12.8	1.4		
		Hi	154.4 ±25.9	151.9 ± 26.4	−2.5		146.4 ± 23.9	146.9 ± 23.9	0.5			146–9 ± 24.1	145.6 ± 24.4	−1.3		
Back Squat (rel)	[%]	Ad	132 ± 11	133 ± 9	1		132 ± 10	133 ± 8	1			133 ± 10	135 ± 12	2		
		Hi	176 ± 20	173 ± 22	−3		168 ± 21	169 ± 20	1			167 ± 20	167 ± 20	0		
Bench Press	[kg]	Ad	84.7 ± 17.3	84.7 ± 17.8	0.0		85.0 ± 20.6	86. 4 ± 21.2	1.4			85.3 ± 18.5	86.9 ± 20.8	1.6		
		Hi	110.3 ± 20	111.9 ± 19.9	1.6		108.1 ± 20.1	109.2 ± 20.7	1.1			106.4 ± 20.0	108.3 ± 20.5	1.9		
Bench Press (rel)	[%]	Ad	101 ± 17	101 ± 19	0.0		101 ± 18	101 ± 18	0			101 ± 19	102 ± 20	1		
		Hi	125 ± 13	127 ± 13	2		124 ± 15	125 ± 15	1			121 ± 14	124 ± 14	3		
1 Mile-Run	[s]	Ad	432 ± 54	441 ± 53	9		440 ± 57	445 ± 61	5			433 ± 61	443 ± 61	10		
		Hi	428 ± 63	426 ± 54	−2		417 ± 49	422 ± 51	5			426 ± 47	419 ± 44	−7		

Ad = Advanced; Hi = Highly-advanced; Δ = Difference between PRE and POST; Sign. = Level of significance; ES = Effect size (Cohen’s d); rel. = relative to body weight. Levels of significance were only calculated for changes from PRE to POST in the placebo group and marked as follows: *= *p* ≤ 0,05; **= *p* ≤ 0,01; ***= *p* ≤ 0,001). Significant differences between group were marked as follows: # = *p* ≤ 0,05; ##; *p* ≤ 0,01; ### = *p* ≤ 0,001.

### 3.5. Muscle damage – CK and Myo

**Figure 5.**
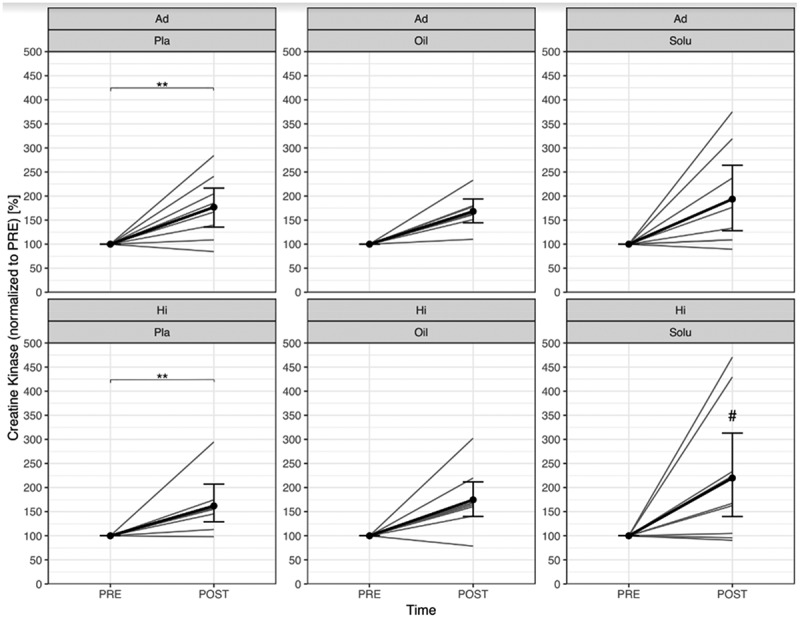
Comparison of normalised creatine kinase concentration from PRE to POST, divided by group and treatment (grey lines represent the individual courses).

The serum concentration of CK increased significantly from PRE to POST in both performance levels (Ad: *p* = 0.002; Hi: *p* = 0.003). This effect was not influenced by CBD-Oil (Ad: *p* = 0.327; Hi: *p* = 0.614). In the Hi group, a significant group difference (stronger increase) was found between CBD-Solu and PLA (*p* = 0.049) (Figure 5). The individual differences between pre and post are shown in supplemental material (Figure 5b).

Ad = Advanced; Hi = Highly-advanced; Pla = Placebo; Oil = CBD-Oil; Solu = CBD-Solubilisat. Ad-Pla: *n* = 8; Ad-Oil: *n* = 7; Ad-Solu: *n* = 8; Hi-Pla: *n* = 8; Hi-Oil: *n* = 9; Hi-Solu: *n* = 9.

**Figure 6.**
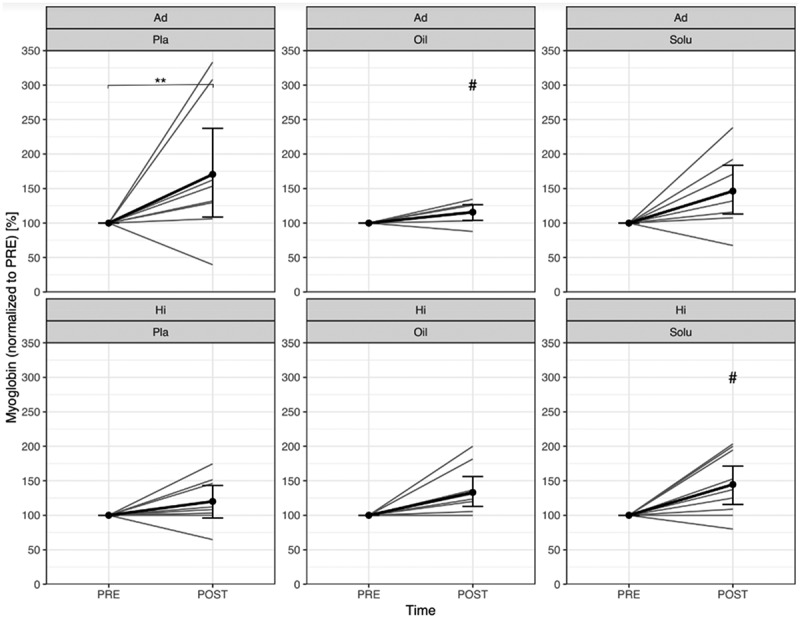
Comparison of normalized myoglobin concentration from PRE to POST, divided by group and treatment (gray lines represent the individual courses).

The myoglobin concentration increased significantly only in the Ad group (p = 0.001), but not in the Hi group (p = 0.220). CBD-Oil significantly reduced the increase in the Ad group (p = 0.049), but not in the CBD-Solu group (p = 0.107). In the Hi group, CBD-Oil showed no significant influence. Influence (p = 0.815), whereas in the CBD-Solu group, a significantly higher increase could be found concerning PLA (p = 0.033) Figure 6). The individual differences between pre and post are shown in supplemental material (Figure 6b).

Ad = Advanced; Hi = Highly-advanced; Pla = Placebo; Oil = CBD-Oil; Solu = CBD-Solu. Ad-Pla: *n* = 8; Ad-Oil: *n* = 7; Ad-Solu: *n* = 8; Hi-Pla: *n* = 8; Hi-Oil: *n* = 9; Hi-Solu: *n* = 9.

### 3.6. Inflammation processes – IL-6 and IL-10

IL-6 shows no significant change over time (Ad: *p* = 0.595; Hi: *p* = 0.975). An influence of CBD-Oil (Ad: *p* = 0.391; Hi: *p* = 0.221) or CBD-Solu (Ad: *p* = 0.786; Hi: *p* = 0.969) could not be found. IL-10 also showed no significant change over time (Ad: *p* = 0.973; Hi: *p* = 0.746). An influence of CBD-Oil (Ad: *p* = 0.919; Hi: *p* = 0.626) or CBD-Solu (Ad: *p* = 0.839; Hi: *p* = 0.442) could also not be detected. The normalized changes and the differences between pre and post are shown in supplemental material (Figure 6C-F).

### 3.7. Oxidative stress – OxLDL and ImAnOx

OxLDL did not change significantly in any group (Ad: *p* = 0.257; Hi: *p* = 0.897) and was not changed by CBD-Oil (Ad: *p* = 0.853; Hi: *p* = 0.586) or CBD-Solu (Ad: *p* = 0.310; Hi: *p* = 0.052). However, there is a trend for a stronger increase in the Hi group with CBD-Solu (*p* = 0.052). ImAnOx also did not change significantly in any group (Ad: *p* = 0.820; Hi: *p* = 0.330). Again, no influence of CBD-Oil (Ad: *p* = 0.960; Hi: *p* = 0.440) or CBD – Solu (Ad: *p* = 0.670; Hi: *p* = 0.540) could be found. The normalized changes and the differences between pre and post are shown in supplemental material (Figure 6G-J).

### 3.8. Immune cell activity – PLR, NLR and SII

**Figure 7.**
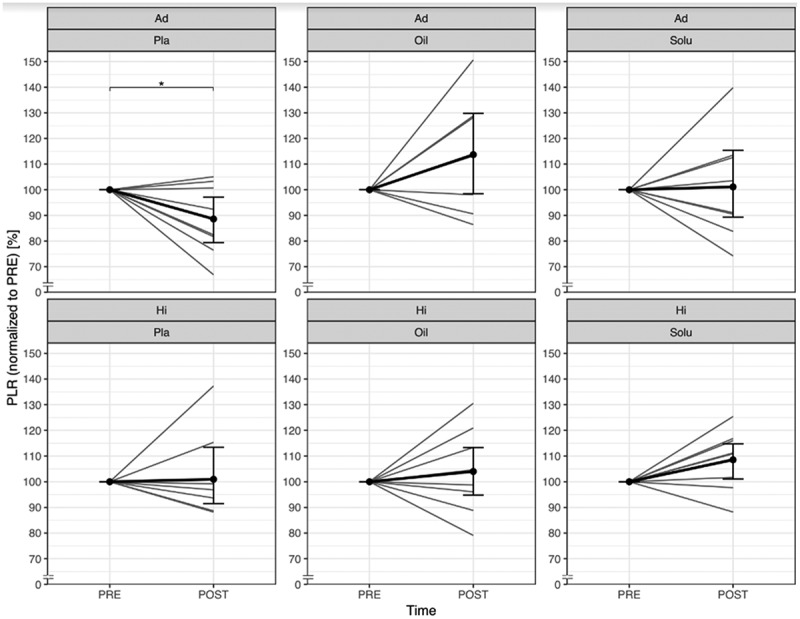
Comparison of normalised platelet-to-lymphocyte-ratio from PRE to POST, divided by group and treatment (grey lines represent the individual courses).

PLR decreased significantly in the Ad group (p = 0.047), but not in the Hi group (p = 0.925). No effect was found for CBD-Solu (Ad: p = 0.556; Hi: p = 0.158), whereas a positive trend was observed for CBD-Oil in both performance levels (Ad: p = 0.083; Hi: p = 0.089). The individual differences between pre and post are shown in supplemental material (Figure 7b)

Ad = Advanced; Hi = Highly-advanced; Pla = Placebo; Oil = CBD-Oil; Solu = CBD-Solu; PLR = platelet-to-lymphocyte-ratio. Ad-Pla: *n* = 8; Ad-Oil: *n* = 7; Ad-Solu: *n* = 8; Hi-Pla: *n*=8; Hi-Oil: *n* = 9; Hi-Solu: *n* = 9.

In NLR, no significant change from PRE to POST could be observed (Ad: *p* = 0.322; Hi: *p* = 0.750). Neither CBD-Oil (Ad: *p* = 0.292; Hi: *p* = 0.558) nor CBD-Solu (Ad: *p* = 0.610; Hi: *p* = 0.190) had a significant effect on the ratio of neutrophils and lymphocytes. For SII also no significant effects over time (Ad: *p* = 0.814; Hi: *p* = 0.752) or by CBD-Oil (Ad: *p* = 0.096; Hi: *p* = 0.652) or CBD-Solu (Ad: *p* = 0.453; Hi: *p* = 0.488) could be found. The normalized changes and the differences between pre and post from both parameters are shown in supplemental material (Figure 7C-F).

### 3.9. Product analyses – CBD-Oil and CBD-Solu

The product analyses of both laboratories showed that both the CBD-Oil and the CBD-Solu contained the specified amount of CBD (60 mg) and did not exceed an amount of THC of 7.1 mg/kg Likewise, only traces of other cannabinoids could be identified full spectrum product.

All biomarker values are summarized in Table 4.

**Table 4.** Means ± standard deviations separated by performance level, as well as differences (Δ) between PRE and POST, levels of significance (sign.) and effect sizes (ES) of all treatments for all biological parameters.

			Placebo				CBD-Oil					CBD-Solu				
Parameter	Unit	Group	PRE	POST	Δ	Sign	PRE	POST	Δ	Sign.	ES	PRE	POST	Δ	Sign	ES
Creatinekinase	[U/l]	Ad	280 ± 163	455 ± 217	175	**	179 ± 74	288 ± 101	109			164 ± 61	297 ± 166	133		
		Hi	269 ± 175	430 ± 282	161	**	308 ± 237	504 ± 373	196			411 ± 427	746 ± 717	335	#	0.66
Myoglobin	[ng/ml]	Ad	38.1 ± 27.9	53.8 ± 33.5	15.7	**	28.9 ± 3.8	33.1 ± 4.4	4.2	#	−0.66	28.0 ± 6.2	39.2 ± 11.8	11.2		
		Hi	37.6 ± 12.5	46.1 ± 25.6	8.5		34.9 ± 12.0	48.0 ± 27.6	13.1			43.7 ± 21.9	64.9 ± 46.3	21.2	#	0.71
Ox LDL	[U/l]	Ad	41.0 ± 18.4	36.5 ± 40.6	−4.5		24.3 ± 15.8	25.7 ± 12.6	1.4			22.6 ± 11.9	40.6 ± 38.5	18.0		
		Hi	25.5 ± 20.9	28.6 ± 31.9	3.1		38.0 ± 33.3	43.0 ± 51.0	5.0			30.8 ± 22.9	59.4 ± 61.8	28.6		
lmANOx	[umol/l]	Ad	293 ± 26	296 ± 11	3		306 ± 29	294 ± 16	−12			288 ± 39	304 ± 38	16		
		Hi	290 ± 32	274 ± 31	−16		297 ± 15	291 ± 37	−6			297 ± 40	269 ± 59	−28		
Il-6	[pg/ml]	Ad	1.24 ± 0.27	1.32 ± 0.33	0.08		1.15 ± 0.29	1.17 ± 0.26	0.02			1.32 ± 0.37	1.36 ± 0.37	0.04		
		Hi	1.17 ± 0.39	1.16 ± 0.45	−0.01		1.24 ± 0.53	1.05 ± 0.34	−019			1.00 ± 0.43	1.26 ± 0.82	0.26		
Il-10	[pg/ml]	Ad	5.24 ± 1.76	5.38 ± 1.76	0.12		5.80 ± 2.91	4.88 ± 2.32	−0.91			1.00 ± 0.43	1.26 ± 0.82	0.26		
		Hi	4.91 ± 2.66	4.98 ± 3.61	0.07		5.74 ± 1.30	5–61 ± 1.93	−0.12			3.96 ± 2.35	6.66 ± 2.67	2.70		
NLR	[AU]	Ad	1.29 ± 0.23	1.23 ± 0.53	−0.06		1.26 ± 0.36	1.49 ± 0.82	0.23			1.58 ± 0.63	1.24 ± 0.37	−0.34		
		Hi	1.31 ± 0.47	1.33 ± 0.47	0.02		1.53 ± 0.54	1.47 ± 0.46	−0.06			1.34 ± 0.29	1.57 ± 0.47	0.23		
PLR	[AU]	Ad	95.0 ± 25.0	83.5 ± 24.4	−11.5	*	84.7 ± 24.4	92.8 ± 18.3	8.1			87.2 ± 21.9	88.3 ± 31.0	1.1		
		Hi	99.2 ± 16.5	99.0 ± 17.8	−0.2		111.2 ± 28.3	115.4 ± 33.9	4.2			104.0 ± 13.0	113.0 ± 18.8	9.0		
SII	[x10^9^/]	Ad	257 ± 52	235 ± 72	−22		252 ± 78	299 ± 156	47			317 ± 156	246 ± 36	−71		
		Hi	320 ± 171	323 ± 185	2		385 ± 207	357 ± 179	−28			316 ± 116	388 ± 175	72		

Ad = Advanced; Hi = Highly-advanced; Δ = Difference between PRE and POST; Sign. = Level of significance; ES = Effect size (Cohen’s d); rel = relative to body weight; AU = Arbitrary unit; Ox LDL = Oxidized Low-Density Lipoprotein; ImAnOx = Total antioxidant status; IL − 6 = Interleukin − 6; IL − 10 = Interleukin − 10; NLR = neutrophil-to-lymphocyte ratio; PLR = platelet-to-lymphocyte ratio; SII = systemic immune-inflammation Index. Levels of significance were only calculated for changes from PRE to POST in the placebo group and marked as follows: * = p ≤ 0,05; ** = p ≤ 0,01; ***= p ≤ 0,001). Significant differences between group were marked as follows: # = p ≤ 0,05; ## = p ≤ 0,01; ### = p ≤ 0,001.

## 4. Discussion

This study aimed to investigate the influence of a short-term chronic CBD application on the recovery process during an intensive training week. It was found that the performance level of the subjects was a decisive factor and that they responded differently to the training protocol and the CBD application. Regardless of the performance level, an increase in biomarkers for muscle damage and a reduction in performance could be induced by the training protocol. Only CBD-Oil was associated with a reduction in MYO concentration in advanced athletes. Moreover, the reduction in performance differs between the two levels of performance. In the ad-group, primarily a reduction in jumping ability was observed, whereas, in the highly advanced athletes, the squat performance was reduced. No clear effect of the two CBD products could be determined on either performance parameter.

Similar to previous studies, muscle damage could be induced by the training protocol [37]. The absolute performance reduction of the highly advanced PLA group also corresponds to the observations of previous studies [37]. However, in contrast to chronic alpha lipoic acid application, no influence of CBD could be detected. Possible reasons for the different effects could be the dosage of the individual supplements and their bioavailability. In a previous study, alpha lipoic acid was supplemented twice daily and consequently in higher doses. Even though the same mechanism of action is suspected for both, they have not yet been sufficiently investigated [78–80]. It can therefore be assumed that possible differences still exist. Additionally, there is currently no evidence to support the use of short-term chronic CBD application to minimize performance decline in highly advanced athletes after multiple intense training sessions. Furthermore, in comparison to previous studies, no negative effect on strength capacity was observed in highly advanced athletes. In a previous pilot study, a greater loss of performance was observed after intensive strength training with CBD application [32]. Due to the different results, further studies with highly advanced athletes are necessary. Higher dosages and potential side effects such as liver and kidney functionality should be investigated. Opposed to highly advanced athletes, no trend of performance decline could be observed in advanced athletes. One possible factor could be the greater development potential of only advanced athletes compared to highly advanced athletes [81]. Although the training loads are relatively adapted to the performance level, it can be assumed that the fatigue symptoms are different for the same training protocol. In addition, due to the slight increase in the squat and bench press over time in all three treatments, it can be assumed that an adaptation effect was induced in advanced individuals. Nevertheless, a decrease in jumping performance was found, confirming the assumption that fatigue to a standardized training protocol is dependent on performance level. In addition, similar to previous studies with advanced athletes, no effects of CBD on the countermovement jump could be observed [32].

Concerning the biomarker for muscle damage a clear increase in both parameters were observed after the intensive training protocol. An inhibitory effect of CBD oil was only observed in the MYO concentration of advanced individuals. This effect could not be observed in any group with CBD-solu. This observation is contrary to the observations after a single intensive strength load with advanced athletes [32]. Although the concentration of the two CBD products were identical, different effects were observed. Both preparations are taken orally. The CBD oil was held in the mouth for 2 min and then ingested, while the solubilisate was drunk directly with water. The different effects could be due to different bioavailabilities. However, this was not tested in this study. A recent review also shows that the intake form of CBD products (e.g. CBD spray, CBD-Oil or smoking) influences bioavailability [82]. However, there are currently no data on the bioavailability of CBD-Solu. Even though different effects were observed after CBD-Oil and CBD-Solu application, no clear difference between the two preparations was noted. Another factor could be a bias in the order of the treatments due to the drop out rate (supplemental material, Table 4). For example, at MYO concentrations, only CBD-Oil showed a significant difference compared to the placebo treatment, but not with CBD-Solu. Among the highly advanced athletes, most of the subjects received the CBD-Solu in the first intervention. However, it was also found that the highest concentrations of the blood parameters were primarily achieved in the first run. Despite a washout period of at least four weeks, this adaptation effect could not be completed antagonized. Nevertheless, no strong effect was observed with any treatment compared to the placebo treatment and currently no clear statements can be made about the effects of short-term chronic application of CBD on muscle damage.

In contrast to the muscle damage parameters, no significant increase was observed in either the inflammatory reactions or the oxidative stress markers after the intensive training week. One reason for this could be primarily anaerobic and high-intensity training impulses. Inflammatory reactions and oxidative stress occur primarily during long-duration and aerobic exercise [83]. In addition, the peak concentrations of inflammatory processes and oxidative stress occur primarily in the first hours after intense exercise. It could therefore be that the period between the last training stimulus and the second blood sample was too far apart. In contrast, a trend of CBD could be observed for PLR. The immune cell activity was reduced by the intensive training week and slightly inhibited by the CBD-Oil. However, the data on the ratios of immune cell activity in the context of sport is very limited [53,84], so the possible effects of CBD-Oil are not clear and cannot be interpreted at this point.

## 5. Limitations

Despite the clear standardization and defined inclusion criteria in this study, it has some limitations. One clear factor is the relative failure rate due to the SARS-CoV-2 pandemic. A total of 8 subjects could no longer reach their performance level due to the side effects of COVID-19 and could not complete the study. Consequently, there was a bias in the order of treatment, which may have an impact on the results. Especially in the highly advanced group, most of the athletes had CBD-Solu in the first intervention phase. It was also found that training-induced muscle damage was greatest in the first intervention phase, resulting in an adaptation effect despite a four-week washout period to the training protocol. Based on this, the stronger increases in individual biomarkers with CBD-Solu and the treatment difference to PL can be explained. To counteract this, the washout phase would probably have to last even longer than four weeks. In addition, the sequence of treatments should be specified in all future studies in order to better explain possible effects. Another limitation of this study is the relatively small sample sizes of the two performance levels. However, there are currently no established guidelines and evidence-based classifications around the interrelationships of food or supplements on regenerative capacity in a sports context. But, this study clearly shows that performance level and exercise protocol have a direct influence on the potential regenerative effects of supplements. Consequently, future studies should also precisely define the performance level of the subjects and declare clear inclusion criteria. As a final limitation of this study, the CBD concentrations in the blood should have been checked to verify possible differences in the bioavailability of CBD-Oil and CBD-Solu. Unfortunately, this was not implementable on an analytical and financial basis.

## 6. Conclusion

This study was the first to investigate the effect of short-term chronic CBD application after an intensive training protocol. Moderate effects of CBD on MYO concentration were found in advanced athletes, but not in highly advanced individuals. The reduced muscle damage could not be transferred to performance parameters. Additional key findings were that the performance level and physiological adaptations to the exercise protocol need to be considered more in future studies. It can be assumed that the effects of supplements are strongly dependent on the performance level and the training protocol.

## Supplementary Material

Supplemental Material

## Data Availability

anonymized raw data can be viewed and obtained upon request to the corresponding author.

## Abbreviations

The following abbreviations are used in this manuscript:

1RM: One-Repetition Maximum
BS: Back Squat
CMJ: Countermovement Jump
CK: Skeletal Muscle Specific Creatine Kinase
Myo: Myoglobin
ln: Logarithm
SE: Standard Error
ES: Effect Size
CBD: Cannabidiol
PLA: Placebo
∆^9^-THC: ∆^9^-tetrahydrocannabinol
NCAA: National Collegiate Athlete Association
FDA: U.S. Food and Drug Administration
WADA: World Anti-Doping Agency
NCSA: National Strength and Conditioning Association
AIC: Akaike Information Criterion
∆: Delta
t: time
NSAIDs: Non-Steroidal Anti-Inflammatory Drugs
NFL: National Football League
RI: Recovery Interval
LME: Linear Mixed Effect Model
DF: Degrees of Freedom
BW: Body weight
