# Supplementary material for: Influence of short-term chronic oral cannabidiol application on muscle recovery and performance after an intensive training protocol - a randomized double-blind crossover study
Source: J Int Soc Sports Nutr. 2024 Apr 4;21(1):2337252. doi: 10.1080/15502783.2024.2337252 (PMC10997358; doi:10.1080/15502783.2024.2337252)
Supplement: Supplemental Material [file RSSN_A_2337252_SM9760.docx]

Supplemental Material


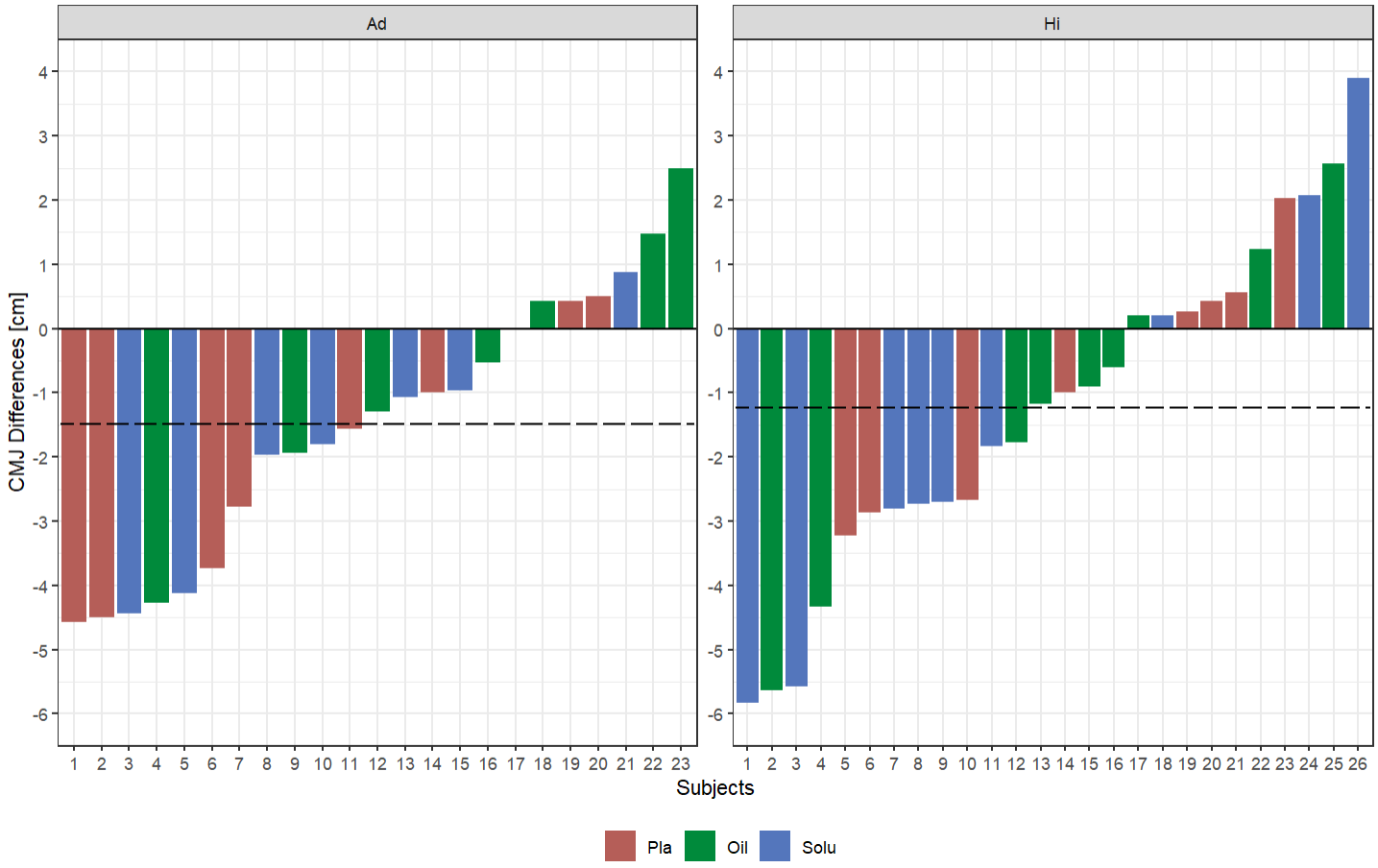

Ad = Advanced; Hi = Highly-advanced; PLA = Placebo; Oil = CBD-Oil; Solu = CBD-Solubilisat.

Figure 3b: Countermovement jump - Differences of all participants as well as the mean value (dotted line)


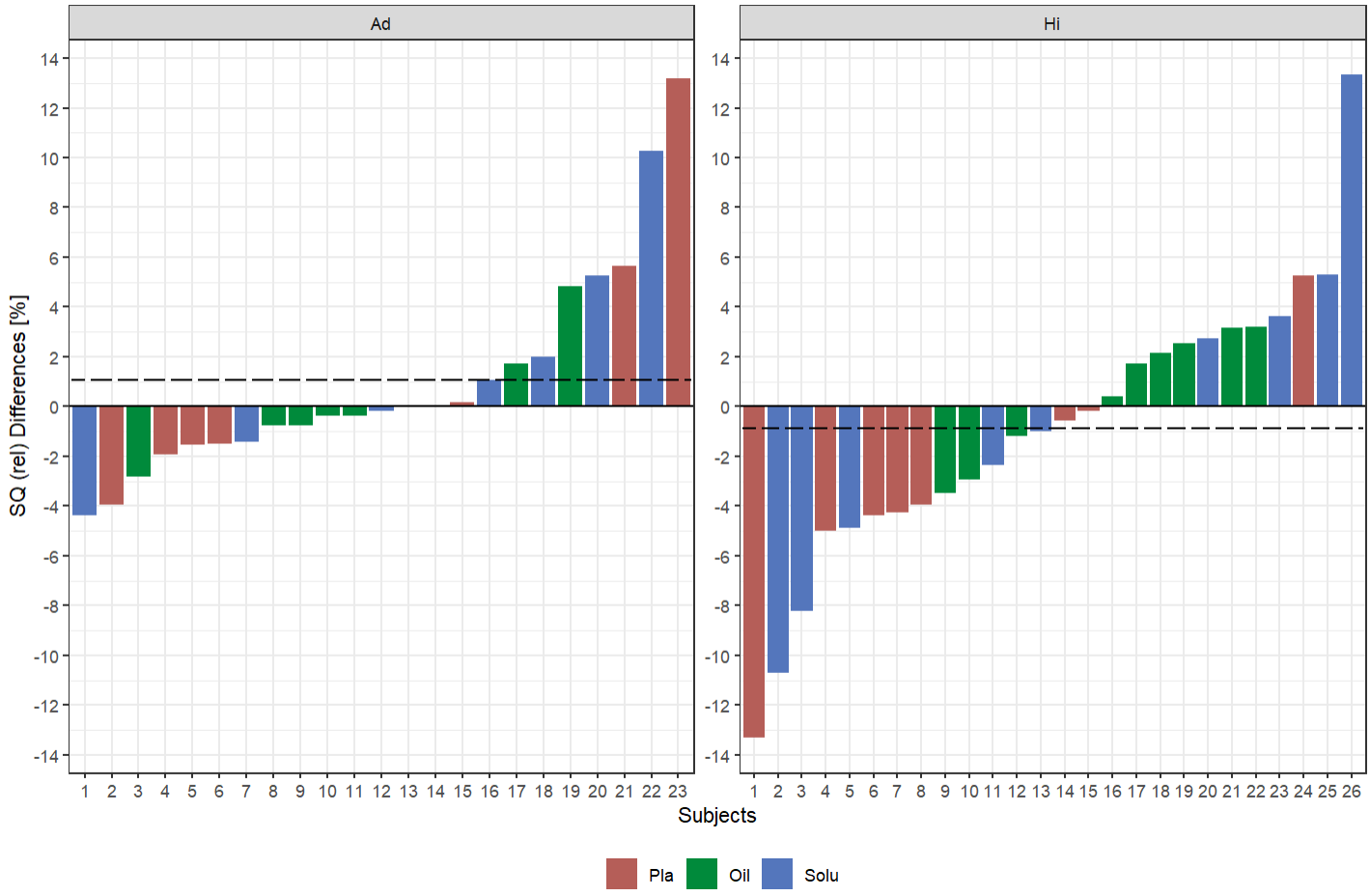

Ad = Advanced; Hi = Highly-advanced; PLA = Placebo; Oil = CBD-Oil; Solu = CBD-Solubilisat; rel = relative to body weight.

Figure 4b: 1-RM back squat differences of all participants as well as the mean value (dotted line).


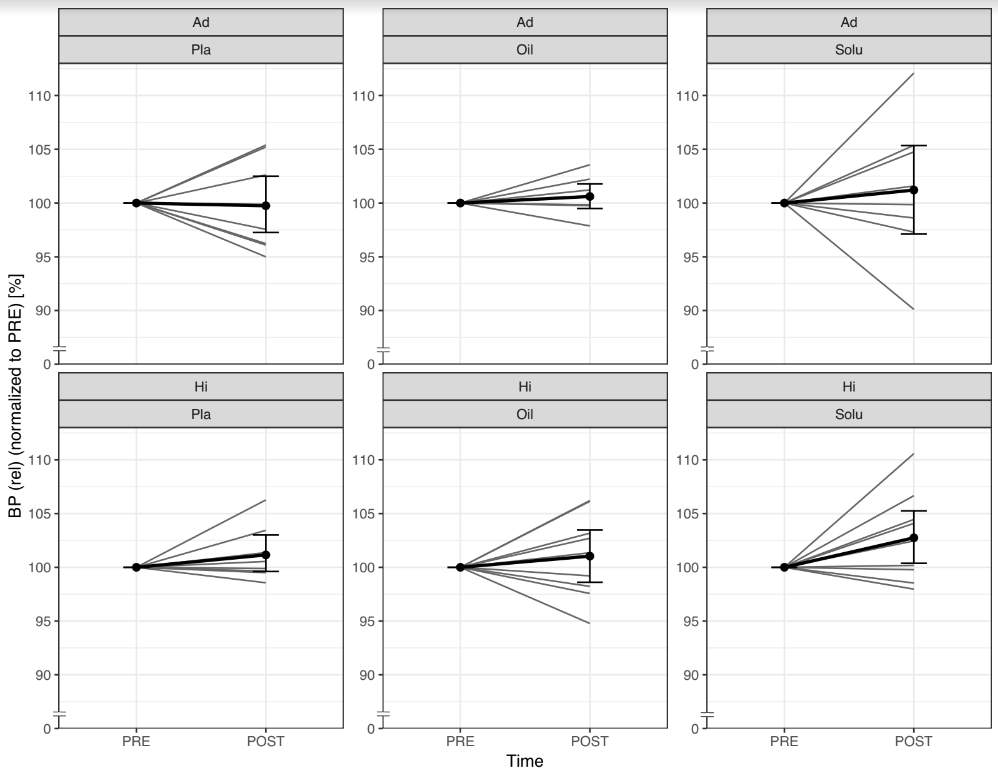

Ad = Advanced; Hi = Highly-advanced; Pla = Placebo; Oil = CBD-Oil; Solu = CBD-Solu. Ad-Pla: n=8; Ad-Oil: n=7; Ad-Solu: n=8; Hi-Pla: n=8; Hi-Oil: n=9; Hi-Solu: n=9.

Figure 4C. Comparison of normalised bench press values from PRE to POST, divided by group and treatment (grey lines represent the individual courses).


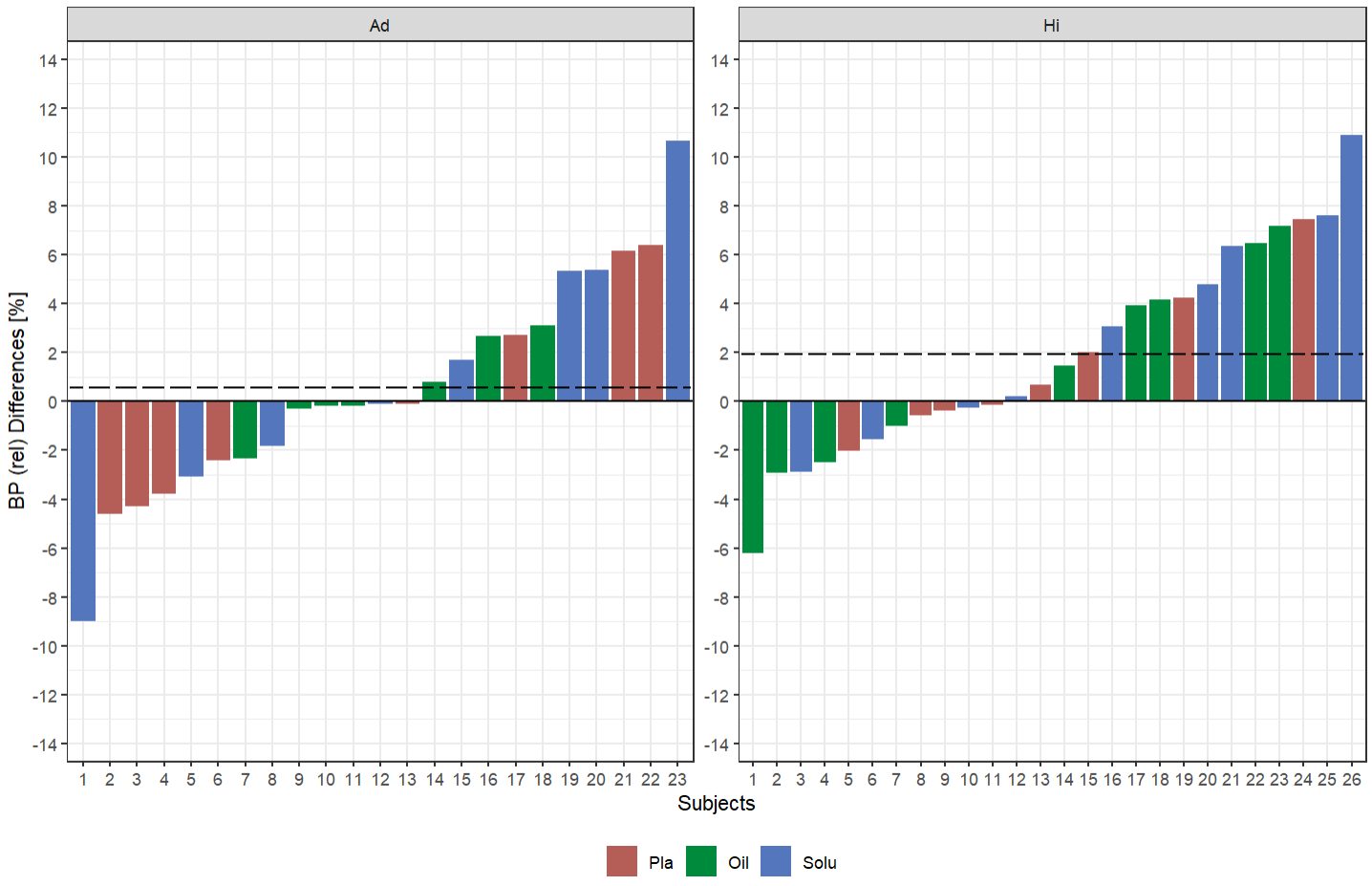

Ad = Advanced; Hi = Highly-advanced; PLA = Placebo; Oil = CBD-Oil; Solu = CBD-Solu; rel = relative to body weight.

Figure 4D: 1-RM bench press differences of all participants as well as the mean value (dotted line).


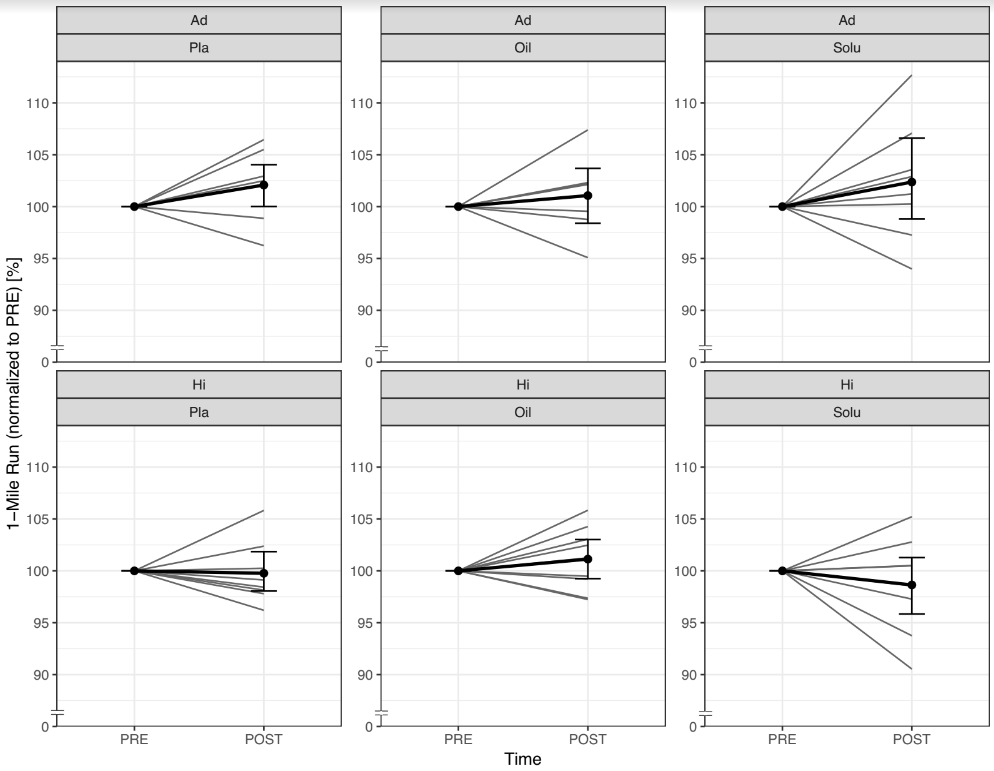

Ad = Advanced; Hi = Highly-advanced; Pla = Placebo; Oil = CBD-Oil; Solu = CBD-Solubilisat. Ad-Pla: n=8; Ad-Oil: n=7; Ad-Solu: n=8; Hi-Pla: n=8; Hi-Oil: n=9; Hi-Solu: n=9.

Figure 4E. Comparison of normalised 1-Mile Run values from PRE to POST, divided by group and treatment (grey lines represent the individual courses).


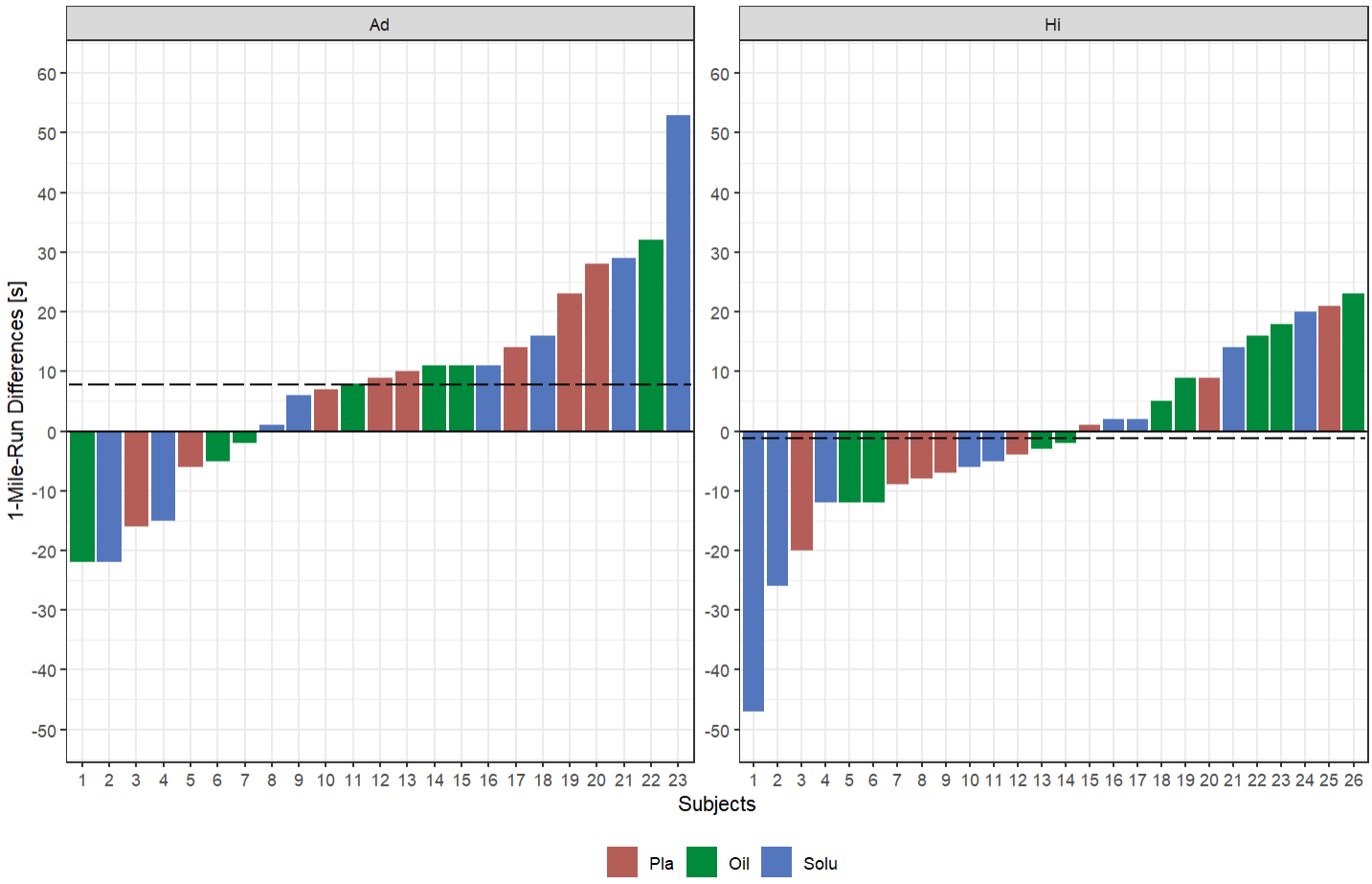

Ad = Advanced; Hi = Highly-advanced; PLA = Placebo; Oil = CBD-Oil; Solu = CBD-Solubilisat; s = seconds.

Figure 4F: 1-Mile Run differences of all participants as well as the mean value (dotted line).


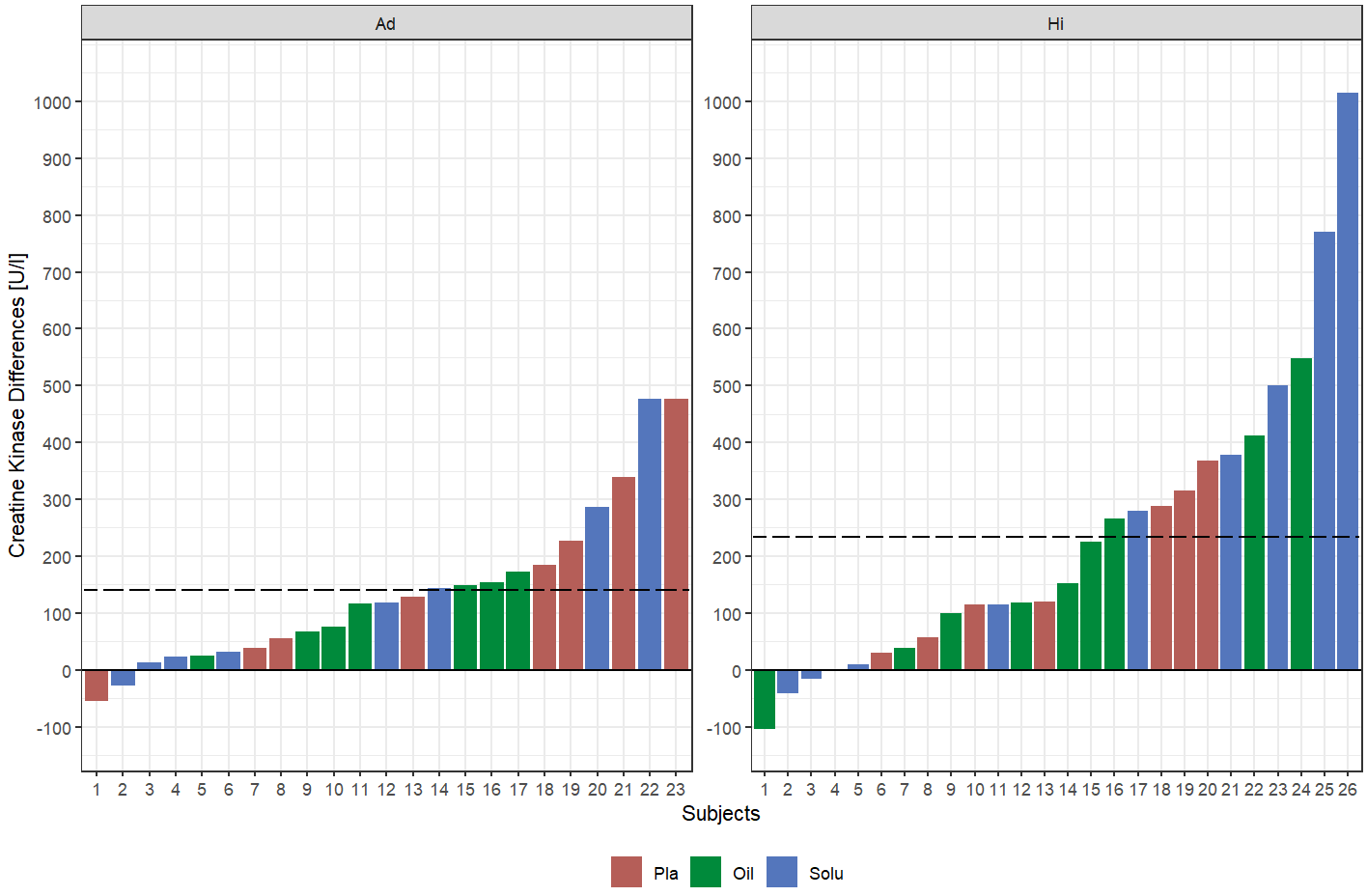

Ad = Advanced; Hi = Highly-advanced; PLA = Placebo; Oil = CBD-Oil; Solu = CBD-Solu.

Figure 5B: Creatine Kinase concentration differences of all participants as well as the mean value (dotted line).


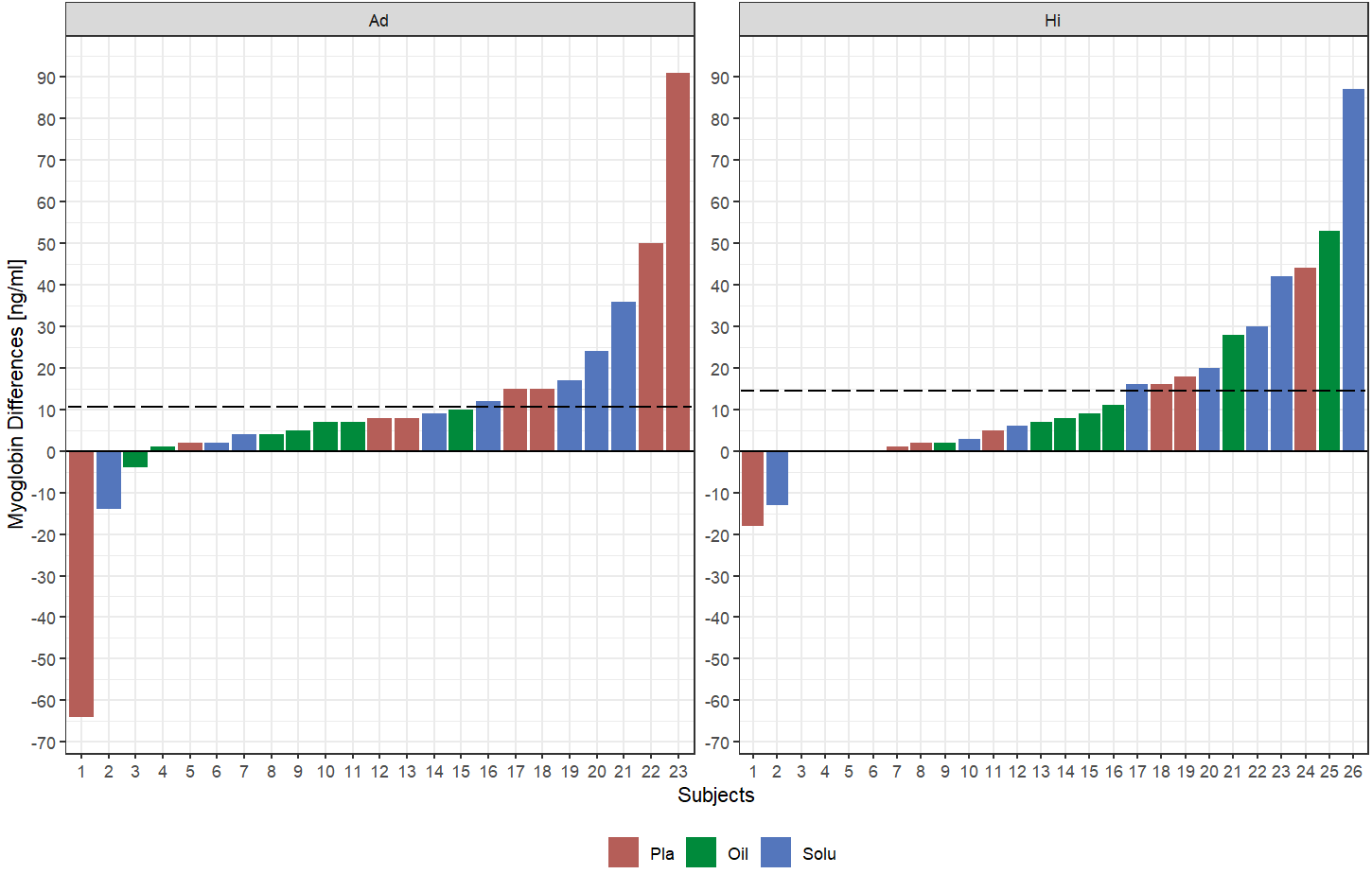


Figure 6B: Myoglobin concentration differences of all participants as well as the mean value (dotted line).


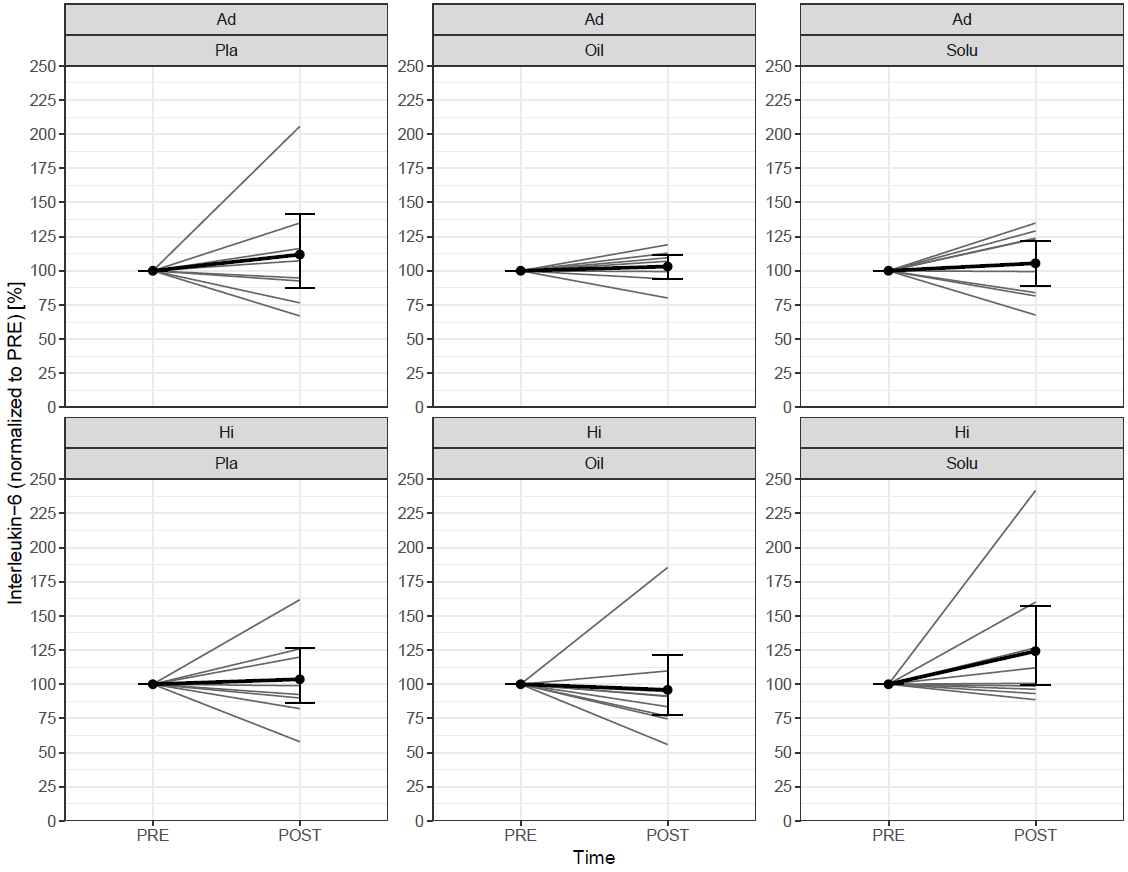


Ad = Advanced; Hi = Highly-advanced; Pla = Placebo; Oil = CBD-Oil; Solu = CBD-Solu. Ad-Pla: n=8; Ad-Oil: n=7; Ad-Solu: n=8; Hi-Pla: n=8; Hi-Oil: n=9; Hi-Solu: n=9.

Figure 6C. Comparison of interleukin 6 values from PRE to POST, divided by group and treatment (grey lines represent the individual courses).


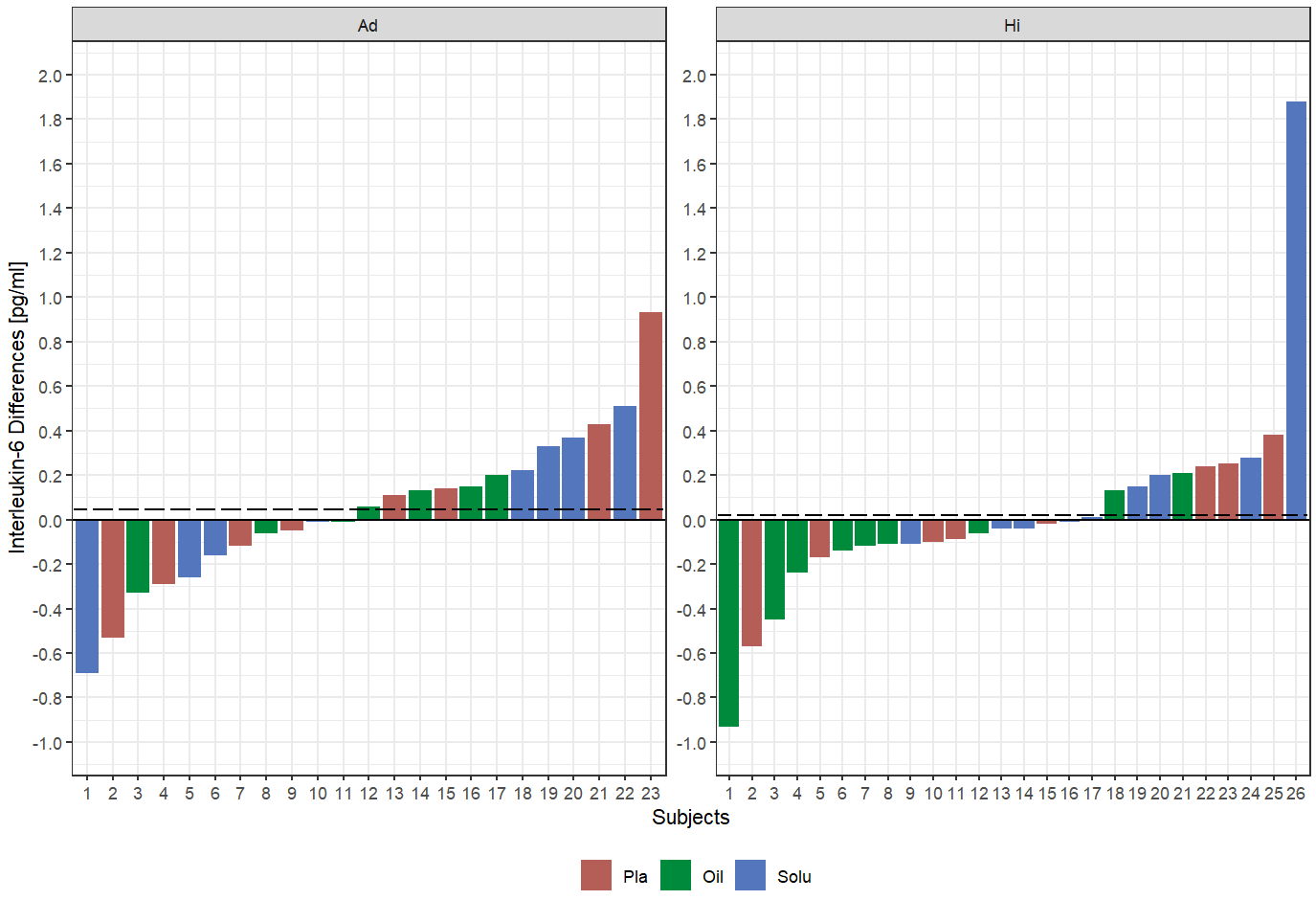

Ad = Advanced; Hi = Highly-advanced; PLA = Placebo; Oil = CBD-Oil; Solu = CBD-Solu.

Figure 6D: Interleukin 6 differences of all participants as well as the mean value (dotted line).


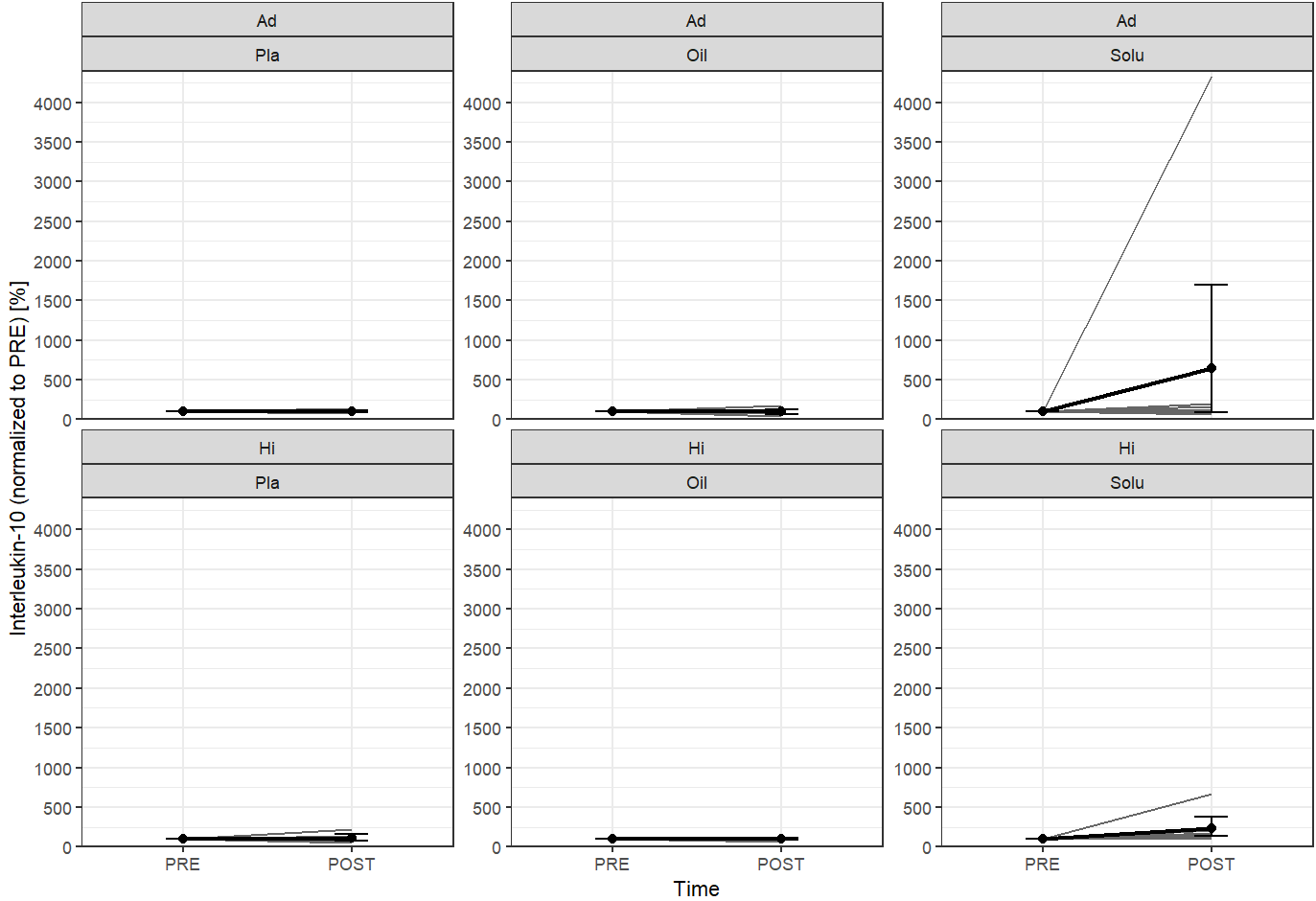


Ad = Advanced; Hi = Highly-advanced; Pla = Placebo; Oil = CBD-Oil; Solu = CBD-Solu. Ad-Pla: n=8; Ad-Oil: n=7; Ad-Solu: n=8; Hi-Pla: n=8; Hi-Oil: n=9; Hi-Solu: n=9.

Figure 6E. Comparison of interleukin 10 values from PRE to POST, divided by group and treatment (grey lines represent the individual courses).


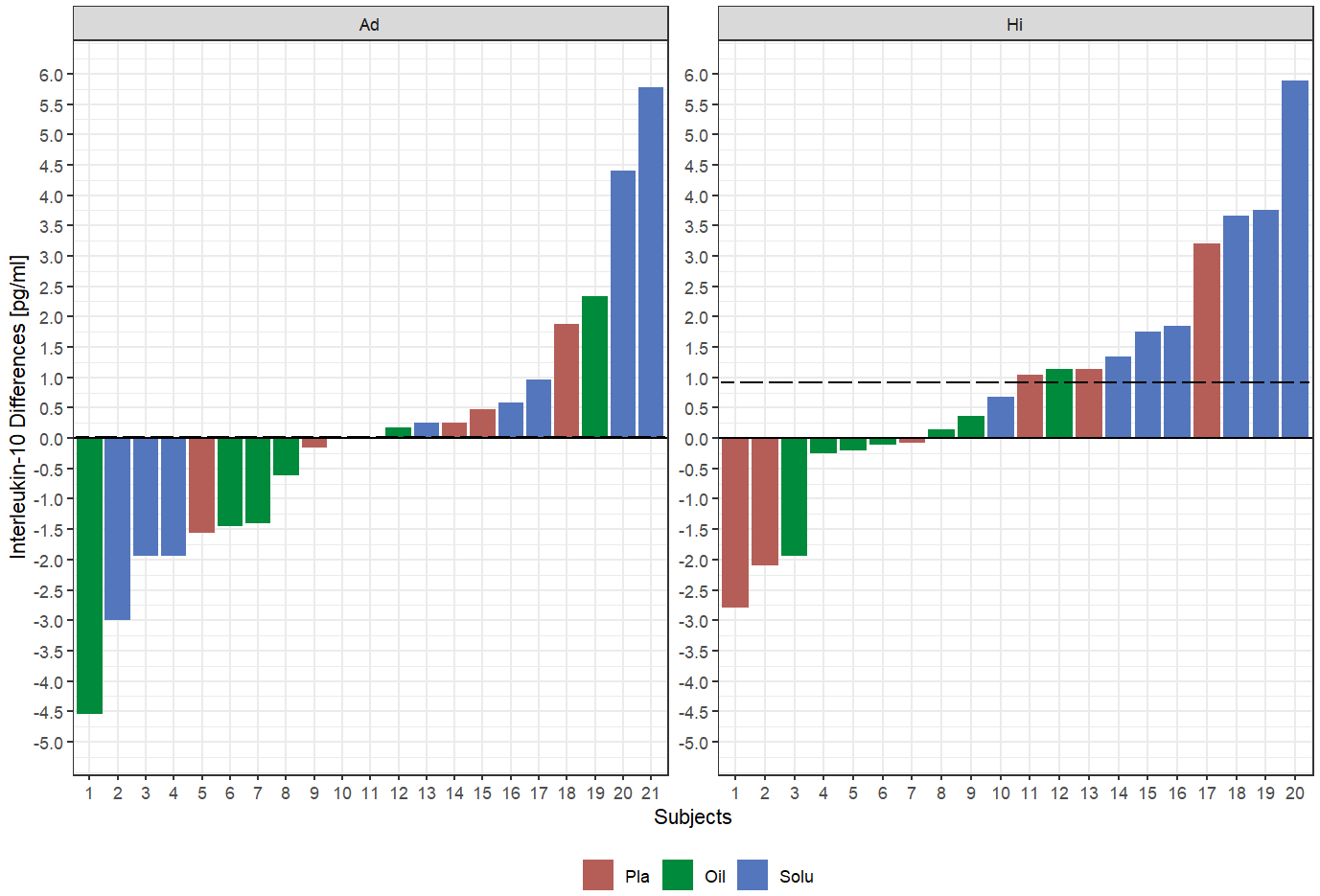

Ad = Advanced; Hi = Highly-advanced; PLA = Placebo; Oil = CBD-Oil; Solu = CBD-Solu.

Figure 6F: Interleukin 10 differences of all participants as well as the mean value (dotted line).


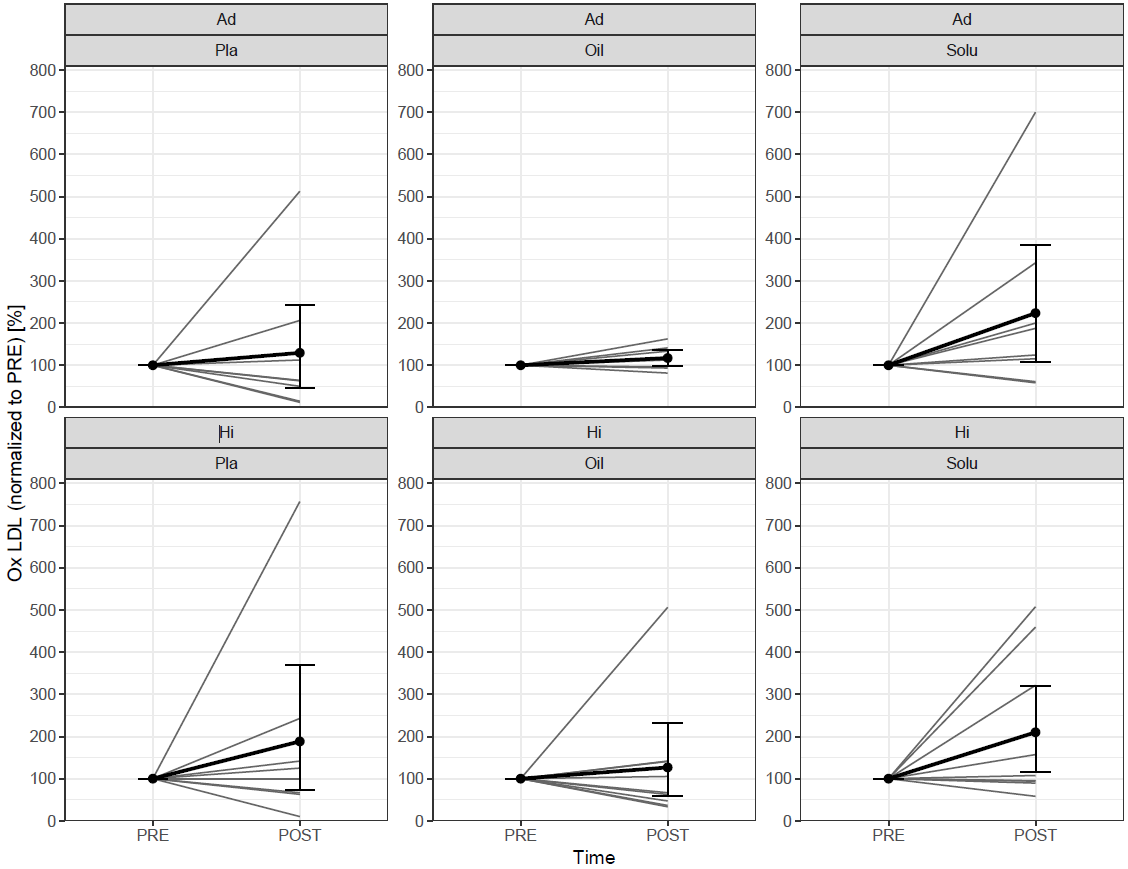

Ad = Advanced; Hi = Highly-advanced; Pla = Placebo; Oil = CBD-Oil; Solu = CBD-Solu; Ox LDL = Oxidized Low-Density Lipoprotein. Ad-Pla: n=8; Ad-Oil: n=7; Ad-Solu: n=8; Hi-Pla: n=8; Hi-Oil: n=9; Hi-Solu: n=9.

Figure 6G. Comparison of OxLDL values from PRE to POST, divided by group and treatment (grey lines represent the individual courses).


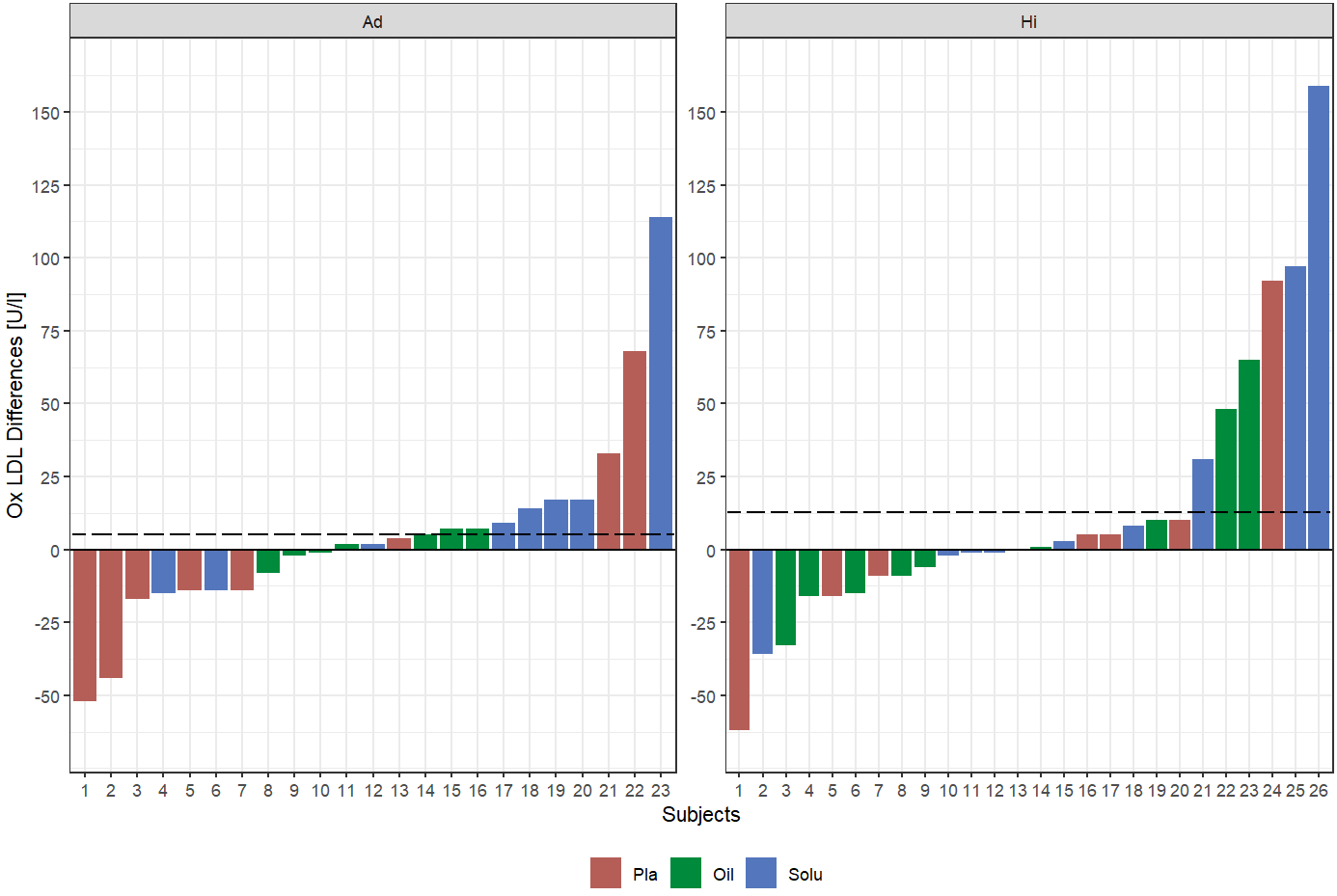

Ad = Advanced; Hi = Highly-advanced; PLA = Placebo; Oil = CBD-Oil; Solu = CBD-Solu; Ox LDL = Oxidized Low-Density Lipoprotein

Figure 6H: OxLDL differences of all participants as well as the mean value (dotted line).


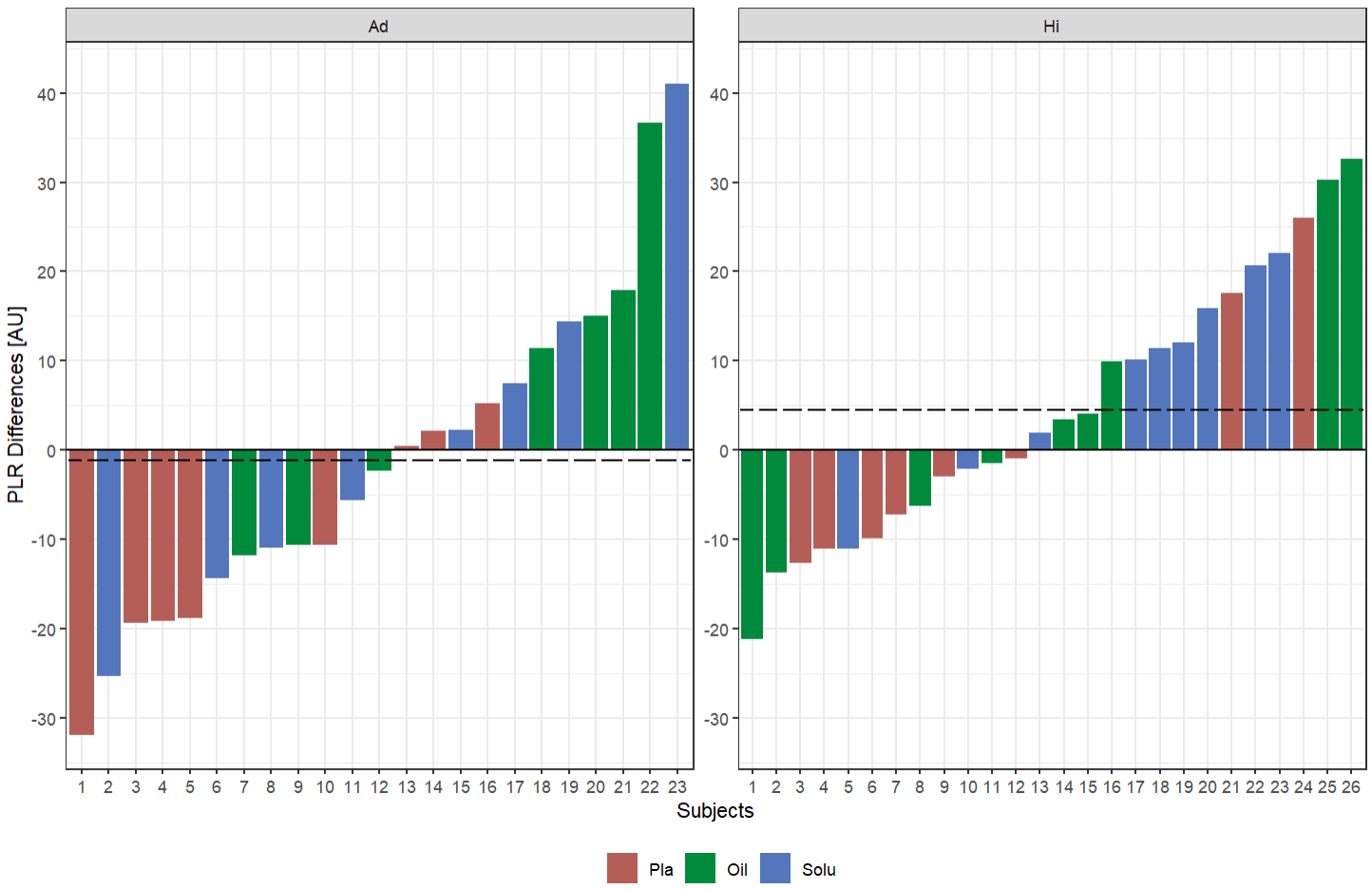

Ad = Advanced; Hi = Highly-advanced; PLA = Placebo; Oil = CBD-Oil; Solu = CBD-Solu; PLR = platelet-to-lymphocyte-ratio.


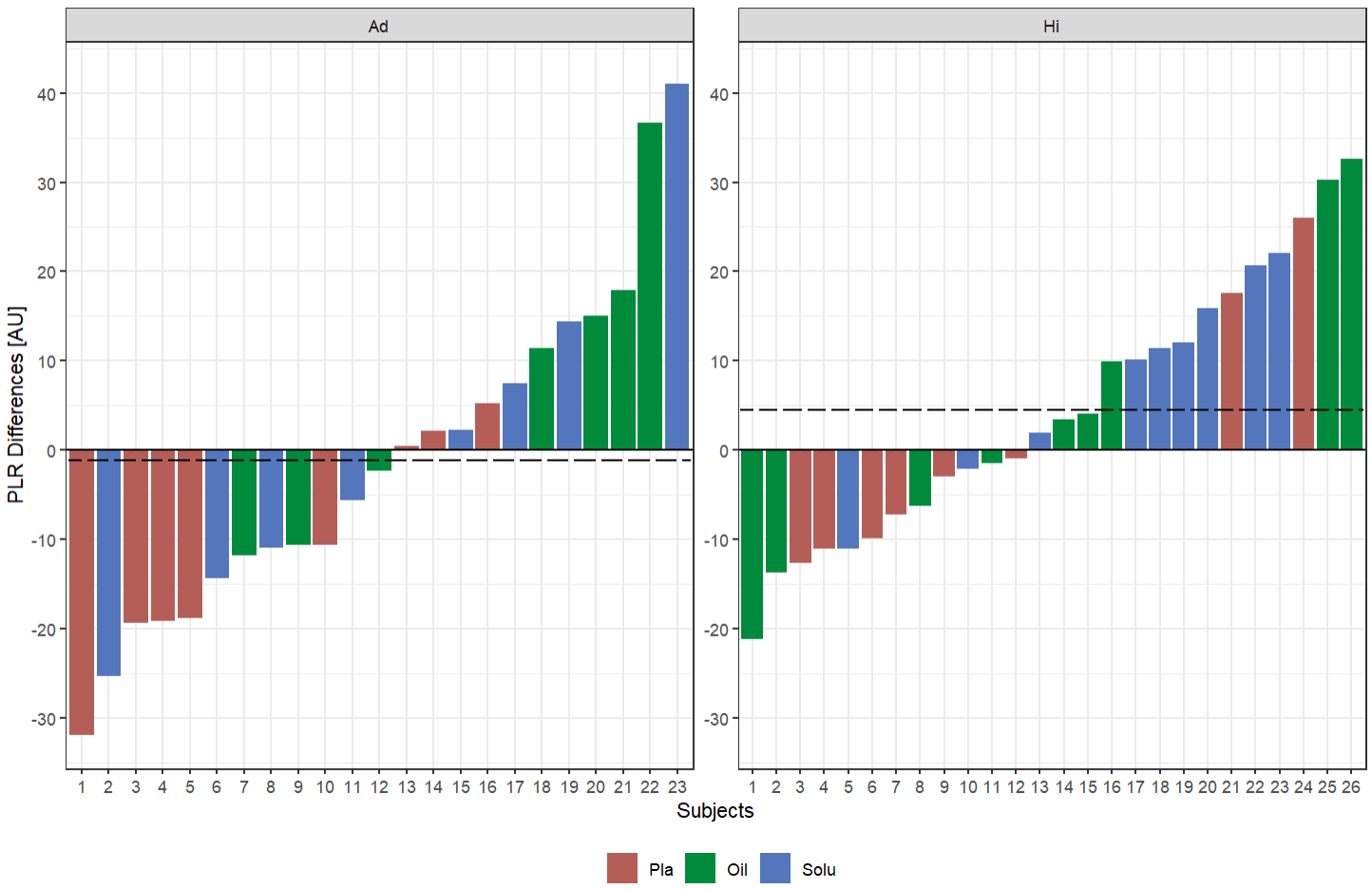

Ad = Advanced; Hi = Highly-advanced; PLA = Placebo; Oil = CBD-Oil; Solu = CBD-Solu; PLR = platelet-to-lymphocyte-ratio.


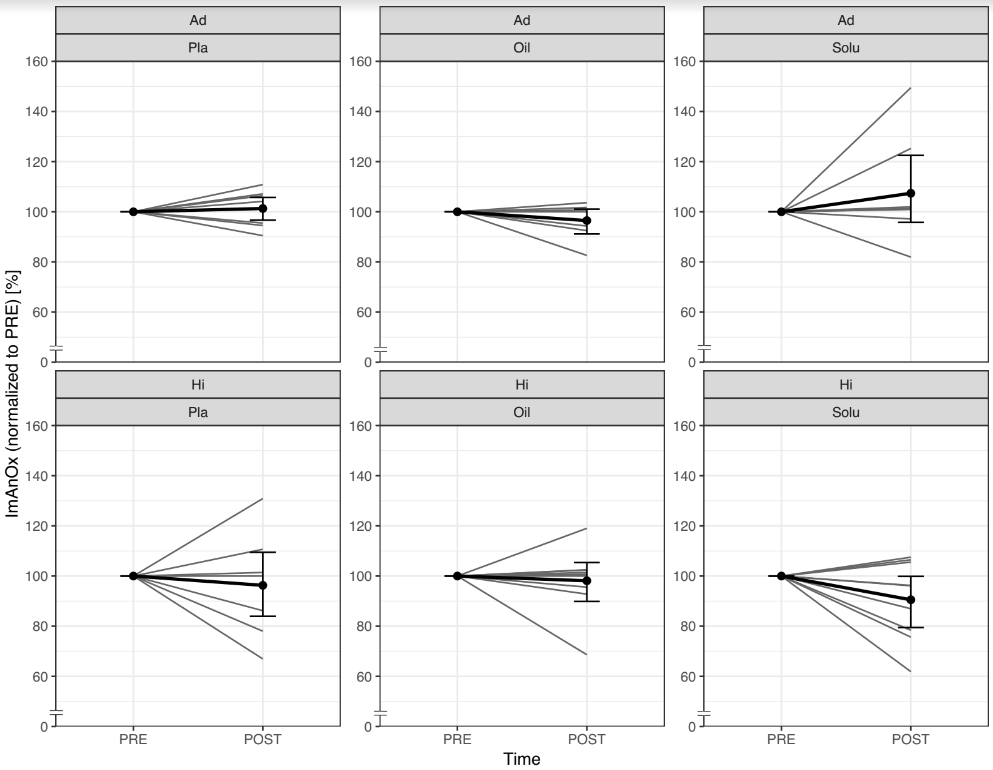

Ad = Advanced; Hi = Highly-advanced; Pla = Placebo; Oil = CBD-Oil; Solu = CBD-Solu; ImAnOx = Total antioxidant status. Ad-Pla: n=8; Ad-Oil: n=7; Ad-Solu: n=8; Hi-Pla: n=8; Hi-Oil: n=9; Hi-Solu: n=9.

Figure 6I. Comparison of ImAnOx values from PRE to POST, divided by group and treatment (grey lines represent the individual courses).


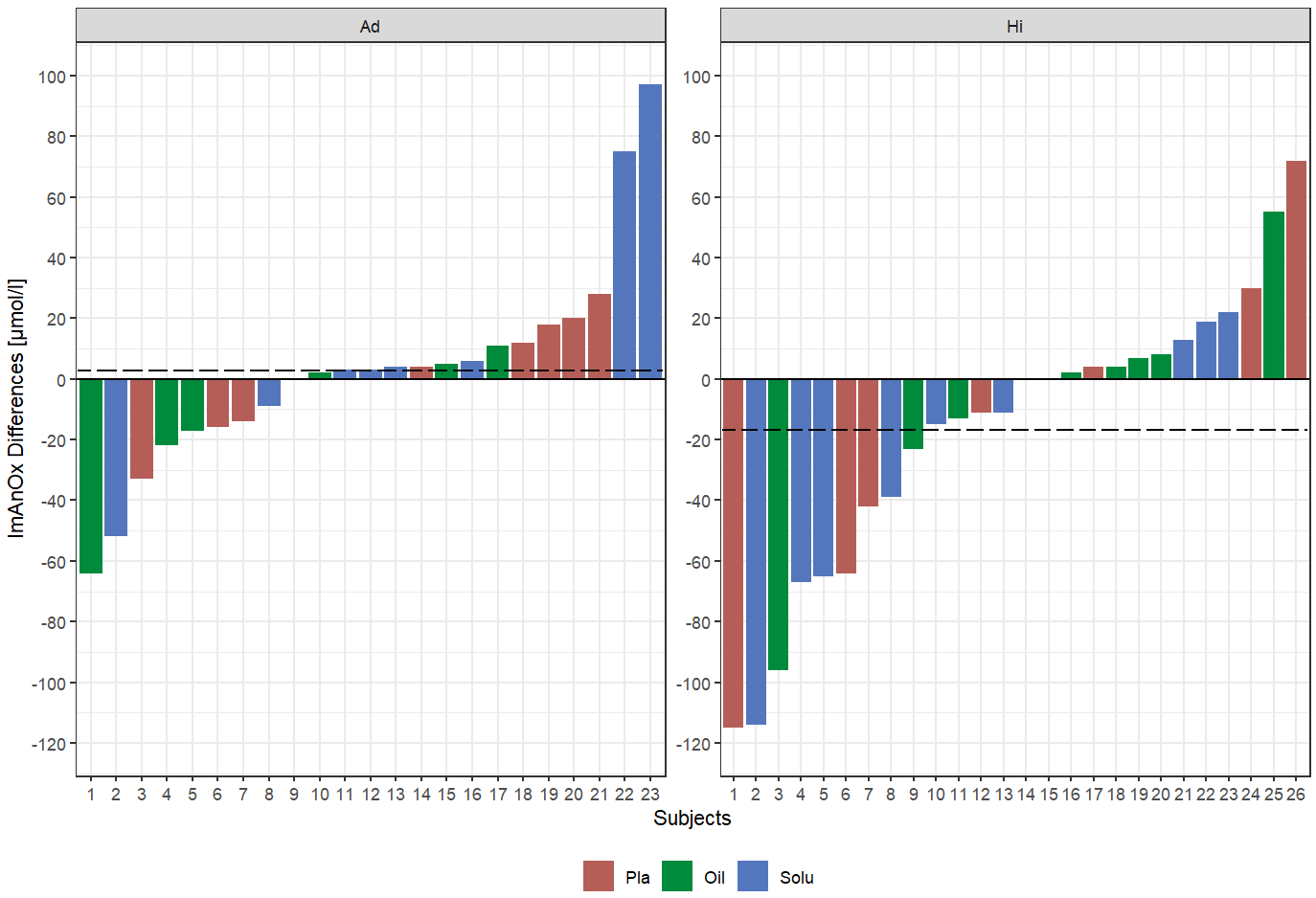

Ad = Advanced; Hi = Highly-advanced; PLA = Placebo; Oil = CBD-Oil; Solu = CBD-Solu; ImAnOx = Total antioxidant status.

Figure 6J: ImAnOx differences of all participants as well as the mean value (dotted line).


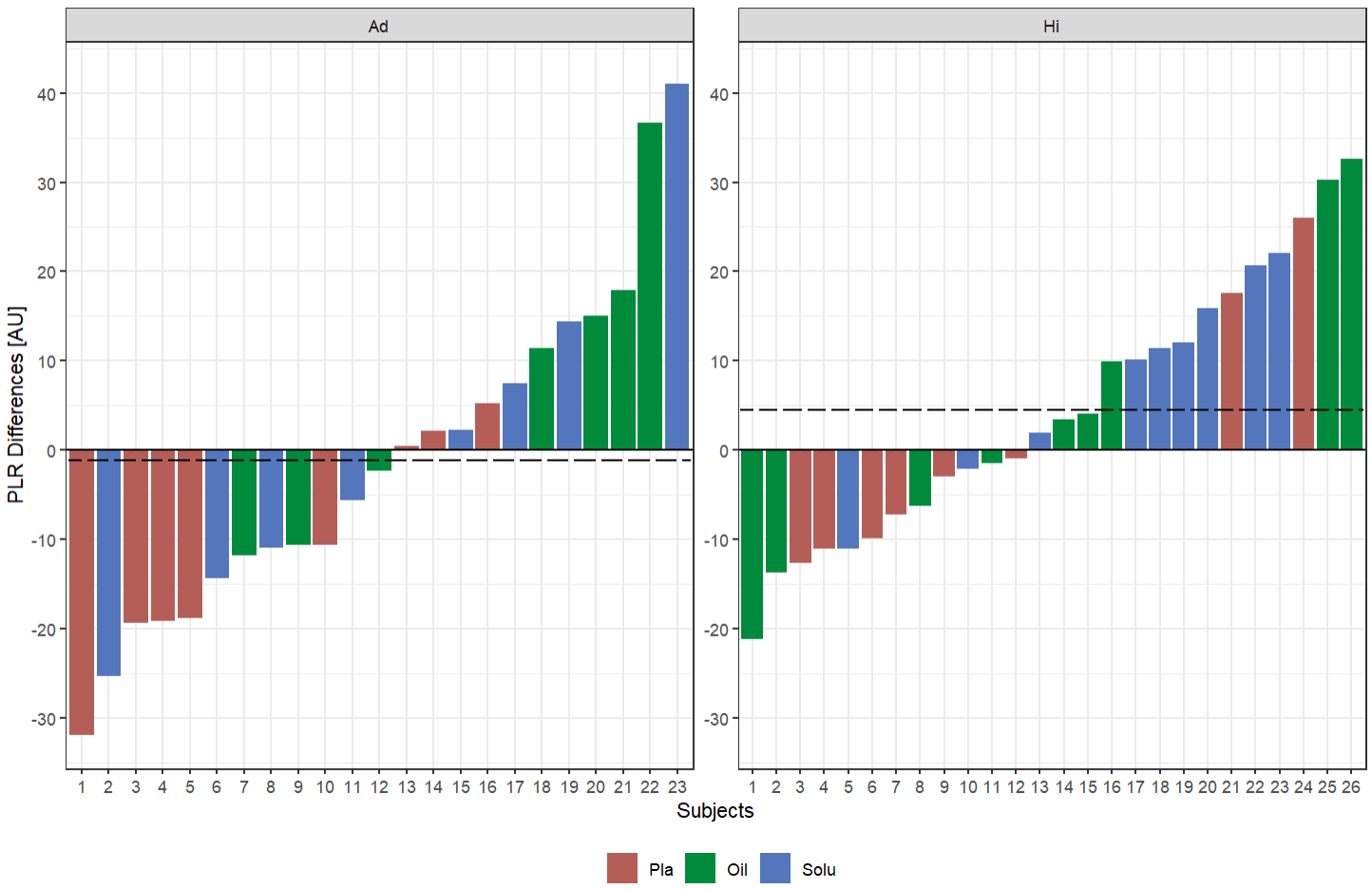

Ad = Advanced; Hi = Highly-advanced; PLA = Placebo; Oil = CBD-Oil; Solu = CBD-Solu; PLR = platelet-to-lymphocyte-ratio.

Figure 7B: Platelet-to-lymphocyte-ratio differences of all participants as well as the mean value (dotted line).


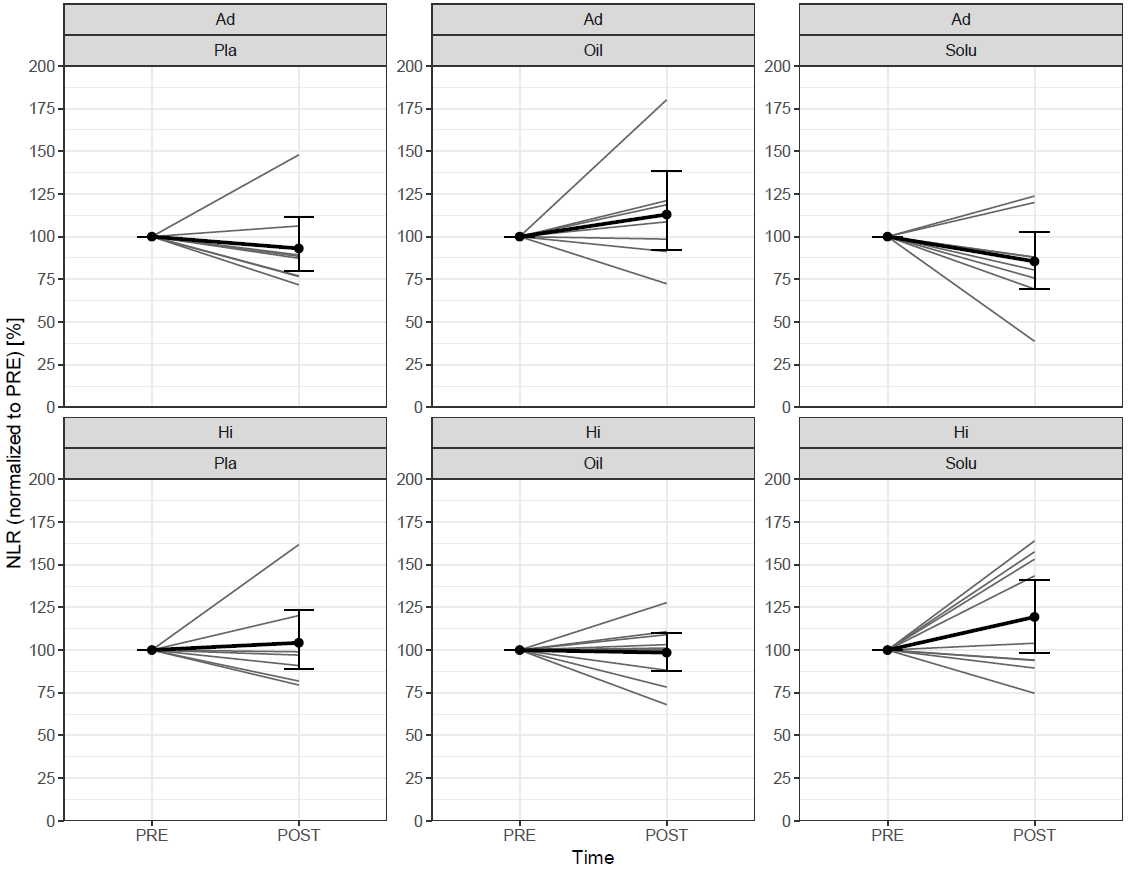

Ad = Advanced; Hi = Highly-advanced; Pla = Placebo; Oil = CBD-Oil; Solu = CBD-Solu; NLR = neutrophils-to-lymphocyte-ratio. Ad-Pla: n=8; Ad-Oil: n=7; Ad-Solu: n=8; Hi-Pla: n=8; Hi-Oil: n=9; Hi-Solu: n=9.

Figure 7C. Comparison of neutrophils-to-lymphocyte-ratio from PRE to POST, divided by group and treatment (grey lines represent the individual courses).


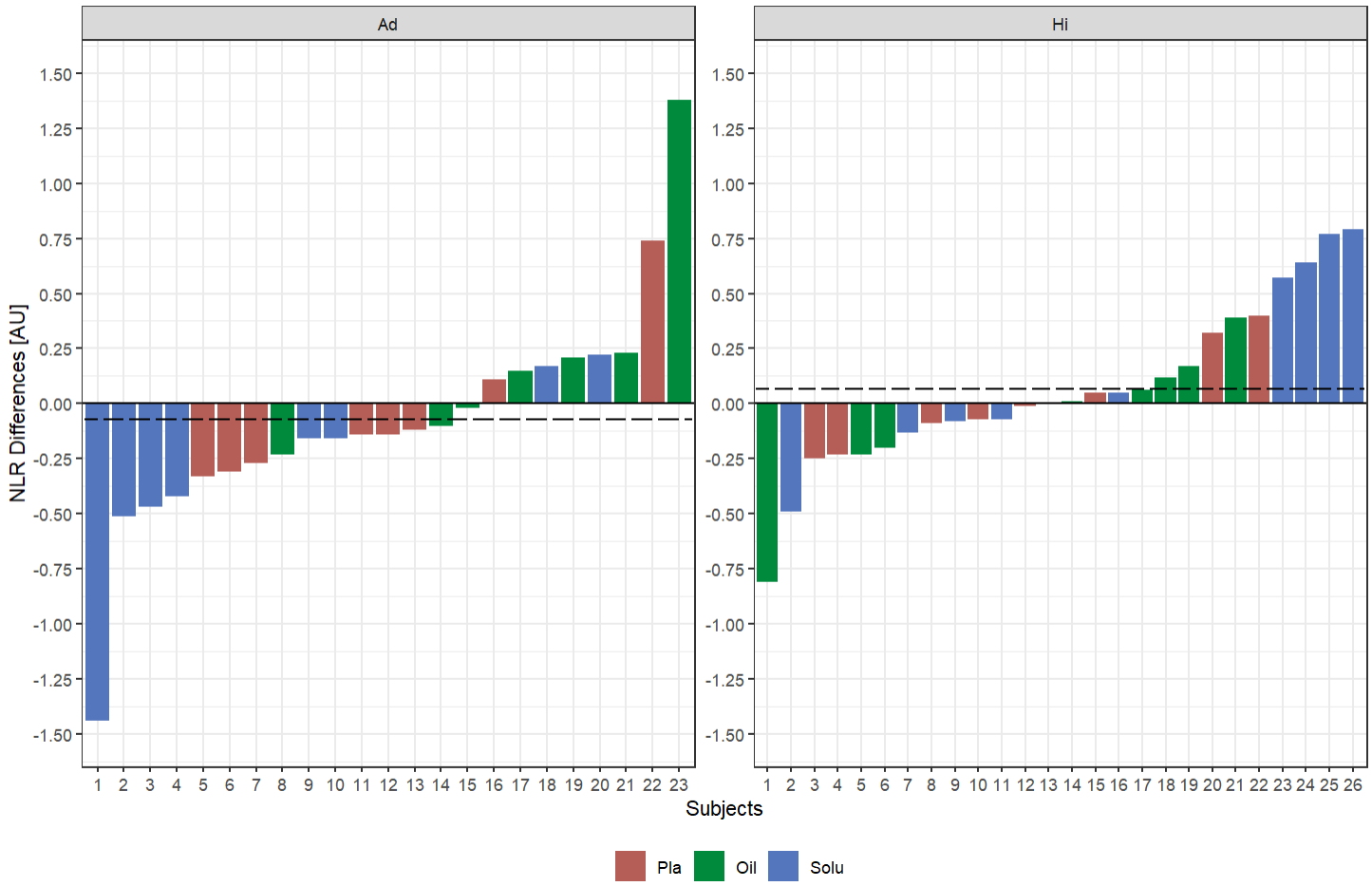

Ad = Advanced; Hi = Highly-advanced; PLA = Placebo; Oil = CBD-Oil; Solu = CBD-Solu; NLR = neutrophils-to-lymphocyte-ratio.

Figure 7D: Neutrophils-to-lymphocyte-ratio differences of all participants as well as the mean value (dotted line).


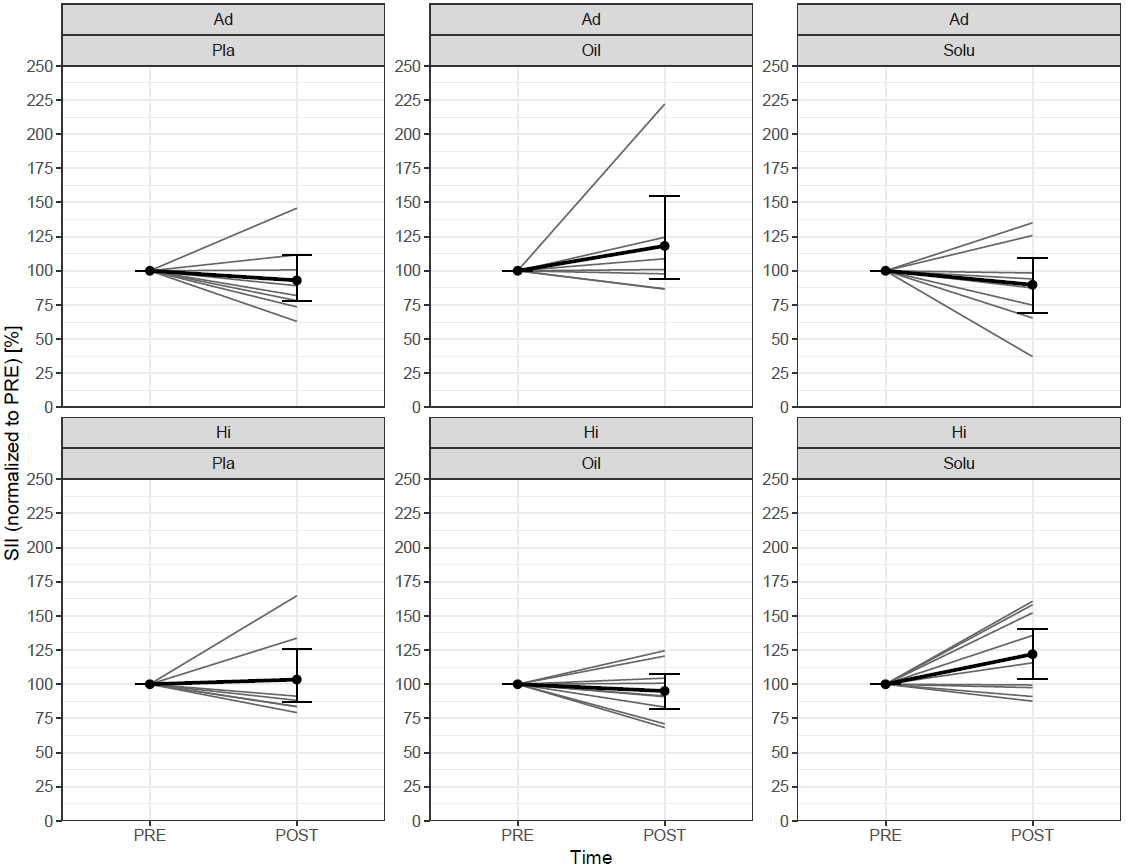

Ad = Advanced; Hi = Highly-advanced; Pla = Placebo; Oil = CBD-Oil; Solu = CBD-Solu; SII = Systemic immune-inflammation index. Ad-Pla: n=8; Ad-Oil: n=7; Ad-Solu: n=8; Hi-Pla: n=8; Hi-Oil: n=9; Hi-Solu: n=9.

Figure 7E: Comparison Systemic immune-inflammation index from PRE to POST, divided by group and treatment (grey lines represent the individual courses).


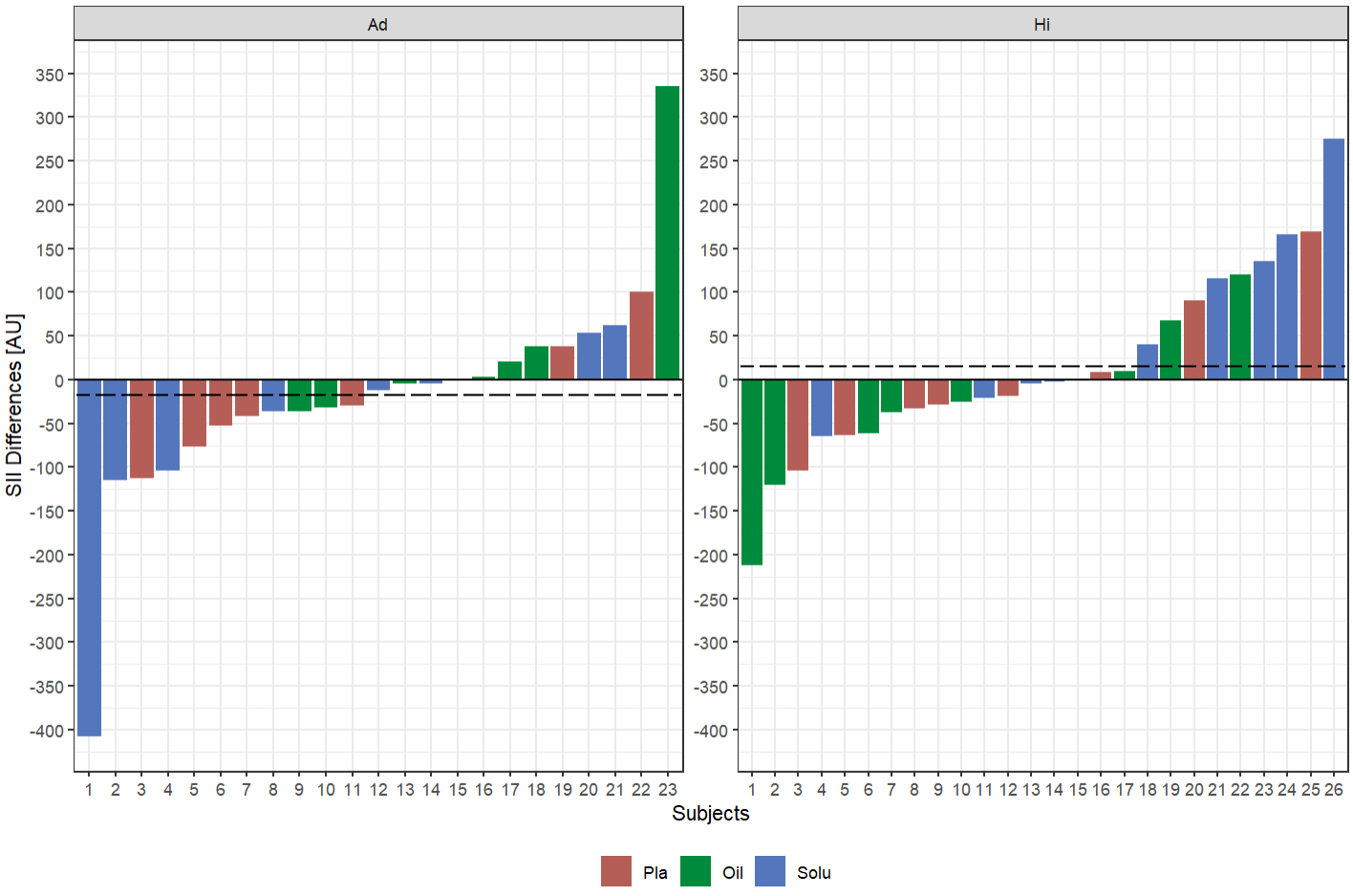

Ad = Advanced; Hi = Highly-advanced; PLA = Placebo; Oil = CBD-Oil; Solu = CBD-Solu; SII = Systemic immune-inflammation index.

Figure 7F: Systemic immune-inflammation index differences of all participants as well as the mean value (dotted line).

Table 4: Order of treatment of the included participants depending on the performance level

| Intervention | Performance Level | Placebo | CBD-Oil | CBD-Solu |
| --- | --- | --- | --- | --- |
| 1 | Ad (n=8) | 5 | 2 | 1 |
|  | Hi (n=9) | 1 | 3 | 5 |
| 2 | Ad (n=8) | 2 | 1 | 5 |
|  | Hi (n=9) | 3 | 5 | 1 |
| 3 | Ad (n=7) | 1 | 4 | 2 |
|  | Hi (n=8) | 4 | 1 | 3 |
